# *Bifidobacterium breve*-derived indole-3-lactic acid ameliorates colitis-associated tumorigenesis by directing the differentiation of immature colonic macrophages

**DOI:** 10.7150/thno.92350

**Published:** 2024-04-22

**Authors:** Yuke Li, Qingxiang Li, Ruoshui Yuan, Yifei Wang, Chuanbin Guo, Lin Wang

**Affiliations:** Department of Oral and Maxillofacial Surgery, Peking University School and Hospital of Stomatology, Beijing, China.

**Keywords:** *Bifidobacterium breve*, colitis-associated tumorigenesis, macrophage differentiation, indole-3-lactic acid, aryl hydrocarbon receptor

## Abstract

**Aim:** To elucidate dynamics and functions in colonic macrophage subsets, and their regulation by *Bifidobacterium breve* (*B. breve*) and its associated metabolites in the initiation of colitis-associated colorectal cancer (CAC).

**Methods:** Azoxymethane (AOM) and dextran sodium sulfate (DSS) were used to create a CAC model. The tumor-suppressive effect of *B. breve* and variations of macrophage subsets were evaluated. Intestinal macrophages were ablated to determine their role in the protective effects of *B. breve*. Efficacious molecules produced by *B. breve* were identified by non-targeted and targeted liquid chromatography-tandem mass spectrometry (LC-MS/MS) analysis. The molecular mechanism was further verified in murine bone marrow-derived macrophages (BMDMs), macrophages derived from human peripheral blood mononuclear cells (hPBMCs), and demonstrated in CAC mice.

**Results:** *B. breve* alleviated colitis symptoms, delayed colonic tumorigenesis, and promoted phenotypic differentiation of immature inflammatory macrophages into mature homeostatic macrophages. On the contrary, the ablation of intestinal macrophages largely annulled the protective effects of *B. breve*. Microbial analysis of colonic contents revealed the enrichment of probiotics and the depletion of potential pathogens following *B. breve* supplementation. Moreover, indole-3-lactic acid (ILA) was positively correlated with *B. breve* in CAC mice and highly enriched in the culture supernatant of *B. breve*. Also, the addition of ILA directly promoted AKT phosphorylation and restricted the pro-inflammatory response of murine BMDMs and macrophages derived from hPBMCs *in vitro*. The effects of ILA in murine BMDMs and macrophages derived from hPBMCs were abolished by the aryl hydrocarbon receptor (AhR) antagonist CH-223191 or the AKT inhibitor MK-2206. Furthermore, ILA could protect against tumorigenesis by regulating macrophage differentiation in CAC mice; the AhR antagonist largely abrogated the effects of *B. breve* and ILA in relieving colitis and tumorigenesis.

**Conclusion:** *B. breve*-mediated tryptophan metabolism ameliorates the precancerous inflammatory intestinal milieu to inhibit tumorigenesis by directing the differentiation of immature colonic macrophages.

## Introduction

Inflammatory bowel disease (IBD), which includes ulcerative colitis (UC) and Crohn's disease (CD), poses a critical hazard for the development of colitis-associated colorectal cancer (CAC) 1. Thus, ameliorating intestinal inflammatory responses has become a potent method to prevent CAC 2, 3.

Colonic macrophages are the abundant immune cell subsets in the lamina propria (LP) that are crucial in regulating gastrointestinal homeostasis 4, 5 and bidirectional regulation of inflammation 6. Most colonic macrophages are replenished by Ly6C^hi^ circulating monocytes with MHCII at a low level 7. As these Ly6C^hi^ monocytes enter the LP, they gradually acquire MHCII and lose Ly6C to differentiate into mature colonic macrophages (MCMs) 7. The differentiation of phenotypes in monocytes coincides with the sustained acquisition of typical macrophage functions, which include maintaining intestinal homeostasis and reducing pro-inflammatory cytokines release 8. However, this process is disrupted by the inflammatory intestinal microenvironment, resulting in a massive accumulation of immature colonic macrophages (ICMs) 8, 9. Under the inflammatory intestinal milieu, conditional pathogenic bacteria, like *Escherichia*, and their metabolites continuously activate ICMs, further aggravating chronic inflammation and leading to tumorigenesis 10. Modulating gut microbiota to direct the differentiation of immature inflammatory macrophages towards a mature homeostatic macrophage phenotype has become a new treatment strategy for CAC 11.

Gut microbiota and their metabolites greatly influence intestinal homeostasis 12, 13. Some species of bacteria, such as *Akkermansia* 14, *Bifidobacterium* 15, and *Lactobacillus* 16, are essential in modulating intestinal immune response. *Bifidobacterium* is among the first residents to colonize the infant's intestinal tract. It can regulate host's immune responses 17, relieve allergic symptoms 18, and treat inflammatory diseases 19. We previously isolated a new *Bifidobacterium* strain named *Bifidobacterium breve* (*B. breve*) *lw01* 20. We demonstrated that oral administration of *B. breve lw01* could regulate the intestinal immune microenvironment by promoting the recruitment of intestinal CD11b^+^CD103^-^ dendritic cells and activating T cells to inhibit subcutaneous tumor growth 21. Recently, the anti-inflammatory properties of *Bifidobacterium* and its associated metabolites, contributing to immune function and development, have been verified 15, 22. However, given the vital role of colonic macrophages in local homeostasis, the underlying molecular mechanisms of *Bifidobacterium* and its metabolites on colonic macrophages need to be further investigated.

In this study, we aimed to elucidate the role of *B. breve lw01* in modulating the dynamics of macrophage subsets and its role in relieving inflammation and preventing CAC tumorigenesis. We discovered that oral administration of *B. breve lw01* could delay the genesis of CAC by attenuating the inflammatory response of the colon, decreasing colonic infiltration of total macrophages, and promoting the differentiation of ICMs. The tumor-suppressive effect was ascribed to indole-3-lactic acid (ILA), a metabolite of L-tryptophan generated by *B. breve lw01*, which could activate aryl hydrocarbon receptor (AhR) in macrophages to regulate their differentiation. Our study provides significant insights into the mechanism by which probiotics blunt tumorigenesis and shall broaden our strategies to leverage microbial treatments to reconstruct immune homeostasis for inhibiting CAC.

## Methods

### Animal experiments

Male C57BL/6 mice (6 weeks old) were obtained from Beijing Vital River Laboratory Animal Technology Co., Ltd. All animals were housed in a specific pathogen-free (SPF) environment with a temperature of 22 ± 2 °C, relative humidity of 50 ± 1%, and a light/dark cycle of 12/12 h. All studies involving animals, including mice euthanasia, complied with Peking University institutional animal care regulations and were conducted according to the AAALAC and the IACUC guidelines (LA2021418).

All mice were acclimatized for one week to standardize the gut microbiota. Five mice were randomly assigned to each cage. The CAC model was established by intraperitoneal injection (i.p.) with azoxymethane (AOM, Sigma-Aldrich) at an initial dose of 10 mg/kg body weight. A week later, animals were given drinking water containing 2% dextran sodium sulfate (DSS, MP Biomedicals) for 7 days and then normal drinking water for 14 days. The cycle was repeated twice. PBS (100 μL) was administered daily by oral gavage. *B. breve lw01* or *Bifidobacterium animalis* (*B. animalis*) *CICC 24672* (1×10^9^ colony-forming units (CFU) in 100 μL PBS) was administered daily by oral gavage. For intestinal macrophage depletion, mice received one cycle of DSS treatment. Clodronate liposomes (CLDs, FormuMax) (100 μL) were intravenously injected (i.v.) every three days. To explore the molecular mechanism, mice received ILA (MedChemExpress) 20 mg/kg/daily by oral gavage and/or the AhR antagonist CH-223191 (Selleck) 10 mg/kg/every two days by i.p.

During the experiment, the body weight of mice was monitored twice a week. After euthanasia, the entire colons, mesenteric lymph nodes (MLNs), and spleens were excised, the colon length was measured, and the number and size of colon neoplasms were quantified, as previously described 23.

### Preparation of *B. breve lw01* and *B. animalis CICC 24672*

*B. breve lw01* was isolated by our group previously 20, and *B. animalis CICC 24672* was purchased from the China Center of Industrial Culture Collection. The two strains were cultured in de Man, Rogosa and Sharpe (MRS) medium (Land Bridge) containing 20 g/L raffinose (Solarbio) and 2.5 g/L L-cysteine (Sigma-Aldrich) at 37 °C under an anaerobic environment. The cultures were resuspended in PBS at a final concentration of 1×10^9^ CFU/100 µL.

### Isolation and culture of murine bone marrow-derived macrophages (BMDMs)

Murine BMDMs were differentiated from tibial and femoral bone marrow cells of male mice aged 6 weeks. Cells were cultured in Roswell Park Memorial Institute-1640 (RPMI-1640) medium (Gibco) with 10% fetal bovine serum (FBS, Sigma-Aldrich), 1% penicillin/streptomycin (Gibco), and 30 ng/ml mouse M-CSF (BioLegend) in a humidified atmosphere containing 5% CO_2_/95% air at 37 °C for 5 days to differentiate to murine BMDMs. Medium was replenished every 2 days. After 5 days, adherent cells containing > 95% CD11b^+^F4/80^+^ macrophages were harvested.

Murine BMDMs were first stimulated for inflammatory macrophage differentiation with 100 ng/mL lipopolysaccharide (LPS, Sigma-Aldrich) and then treated with 500 or 1000 μM ILA in the ILA group. In rescue experiments, murine BMDMs were stimulated with 100 ng/mL LPS, 10 μM CH-223191 or 2 μM MK-2206 (an AKT inhibitor, Selleck), and then treated with 500 or 1000 μM ILA.

### Culture of murine cell lines

CT26.WT and MC38 murine cell lines, purchased from the National Infrastructure of Cell Line Resource, were cultured in Dulbecco's modified Eagle medium (DMEM, Gibco) with 10% FBS and 1% penicillin/streptomycin in a humidified atmosphere containing 5% CO_2_/95% air at 37 °C. The cell lines were treated with 500 or 1000 μM ILA in the ILA group.

### Isolation and culture of macrophages derived from human peripheral blood mononuclear cells (hPBMCs)

The hPBMCs were isolated from healthy donors by gradient centrifugation using the hPBMCs Extraction Kit (Solarbio). Cells were cultured using 30 ng/ml human M-CSF (BioLegend). After 7 days, adherent cells containing > 95% CD11b^+^CD68^+^ macrophages were harvested and treated the same as murine BMDMs. The Ethics Committee of Peking University School and Hospital of Stomatology approved the current study (PKUSSIRB-202275069).

### Flow cytometry

Colonic tissues were dissected, and the fatty portions were discarded. Tissues were then washed with PBS and predigested in PBS containing 10 mM HEPES (Solarbio), 1 mM dithiothreitol (Sigma-Aldrich), and 30 mM EDTA (Solarbio). The pretreated tissues were cut into small pieces and digested with 300 U/mL collagenase VIII (Sigma-Aldrich), 0.15 mg/mL DNase I (Sigma-Aldrich), and 10% FBS in RPMI-1640 medium, filtered through a 70 µm strainer and centrifuged to collect cells. The pellets were resuspended, and the LP immune cells were isolated by gradient centrifugation using Percoll (Solarbio).

MLNs and spleens of mice were mechanically dissociated, and the spleens were then lysed with red blood cell lysis buffer (Solarbio). The single-cell solution was subjected to filtration through a nylon membrane and centrifugated.

Murine BMDMs and macrophages derived from hPBMCs were treated as mentioned above and then collected to label the corresponding markers.

After filtration, isolated cells were resuspended in FACS buffer and Fc-γ receptors were blocked with TruStain FcX (BioLegend). Cells were stained with antibodies directed against CD45, CD11b, F4/80, Ly6G, Siglec-F, CD11c, MHCII, Ly6C, CD3, CD4, CD25, CD8, CD68, CD86, and CD206. Staining with 7-AAD was used to exclude dead cells. BD FACSymphony S6 was used to perform flow cytometry. FlowJo_v10.8.1 was used for data analysis. Detailed information on all antibodies is shown in Table S1.

### Histology, immunohistochemistry (IHC) and fluorescence *in situ* hybridization (FISH)

For histology assessment, 5 μm sections of the distal colon were fixed, treated according to the manufacturer's instructions, and stained with hematoxylin and eosin (H&E). The stained slides were scanned by a microscope (Olympus).

For IHC assays, 5 μm sections were deparaffinized, endogenous enzymes inactivated, antigens repaired, blocked, and incubated with Ki67, phosphorylated STAT3 (p-STAT3), non-phosphorylated β-catenin (activated-β-catenin), F4/80, CD86, CD206, Foxp3, or CD8α antibodies, followed by corresponding secondary antibodies. The stained slides were scanned by a microscope (Olympus). The images were quantified by Image-Pro Plus 6.0. Detailed information on all antibodies is shown in Table S1.

For FISH staining, 5 μm sections were deparaffinized, blocked and hybridized with Cy5-labelled *Bifidobacterium* probe (5'-CATCCGGYATTACCACCC-3', Y represents C or T). Finally, the cell nucleus was counterstained with 4',6-diamidino-2-phenylindole (DAPI, ZSGB-BIO). The stained slides were scanned by a confocal microscope (Leica).

### RNA isolation and quantitative real-time PCR (qRT-PCR)

Total RNA was extracted with the TRIzol reagent (Ambion). The mRNA expression was quantified using SYBR Green (YEASEN) on a qRT-PCR system (ROCGENE). The PCR procedure entailed an initial pre-denaturation step at 95 °C for 10 min, followed by 40 cycles of amplification at 95 °C for 15 s and 60 °C for 1 min. Primer sequences are shown in Table S2.

### Western blotting

Total proteins were extracted from murine BMDMs, macrophages derived from hPBMCs, CT26.WT, and MC38 cell lines using RIPA buffer (Huaxingbio) containing phosphatase and protease inhibitors (Huaxingbio). The proteins were separated on 10% SDS-PAGE and transferred to a PVDF membrane. The membrane was then blocked with 5% non-fat milk and incubated with primary antibodies at 4 °C overnight. Samples were then washed and treated with horseradish peroxidase-conjugated secondary antibodies at room temperature for 1 h. The signals were visualized using an ECL detection reagent (YEASEN). Output images were analyzed using ImageJ. Detailed information on all antibodies is shown in Table S1.

### GMrepo database analysis

Phenotypes associated with the relative abundance of *Bifidobacterium* were acquired from the GMrepo database, which contains a human gut metagenomic data repository for microbiota 24. The relative abundance of *Bifidobacterium* in the feces of healthy individuals, UC, CD, and colorectal cancer (CRC) patients was obtained from the GMrepo database. The data quality was evaluated first, and then the relative abundance of *Bifidobacterium* was analyzed on an online platform.

### Gene expression profiling interactive analysis

The Gene Expression Profiling Interactive Analysis (GEPIA) data source 25 was used to evaluate *CYP1A1*, *CYP1A2*, and *CYP1B1* levels in healthy individuals and CRC patients via the “Expression DIY” page.

### Tumor Immune Single-cell Hub (TISCH) database analysis

The TISCH data source 26 was used to analyze the expression of *CYP1B1* in different cells of CRC patients.

### RNA sequencing

Total RNA of intestinal macrophages, sorted from fresh tissues by flow cytometry, was extracted using the TRIzol reagent. For low-input RNA-Seq library construction, cDNA was amplified by SMART-SeqII combined with a transposase-based library construction technique. First, the rolling cycle amplification was employed to replicate single-stranded circle DNA molecules, and a DNA nanoball (DNB) containing multiple copies of DNA was generated. Combinatorial Probe-Anchor Synthesis allowed sufficient quality DNBs to be loaded into patterned nanoarrays. The sequencing data was filtered with SOAPnuke. Subsequently, clean reads were stored in FASTQ format. Analysis and data mining were performed using Dr. Tom Multi-omics Data mining system.

### 16S rRNA sequencing

16S rRNA was amplified from microbial DNA at the hypervariable region of V3-V4 on the NovaSeq PE250 platform. For high-quality clean tags, raw reads were filtered under specific filtering conditions based on fqtrim_v0.94. Vsearch_v2.3.4 was applied for chimeric sequence filtering. After dereplication, we obtained amplicon sequence variants (ASVs) and operational taxonomic units (OTUs). The final ASV feature table and sequence were used to analyze the microbiota on an online analysis website.

### Liquid chromatography-tandem mass spectrometry (LC-MS/MS) analysis

#### Differential metabolites in CAC mice

The samples were weighted and mixed with an extraction solution. An ultra-high performance liquid chromatography (UHPLC) system (Thermo Fisher) coupled with the Q Exactive HFX mass spectrometer (Thermo Fisher) was then applied. The mzXML format was converted from the raw data by ProteoWizard and then completed with an in-house program, which was developed using R and based on XCMS, for peak detection, extraction, alignment, and integration. Metabolite annotation was performed using an in-house MS2 database (BiotreeDB).

#### Efficacious metabolites in the culture supernatant of *B. breve lw01* (BCS) and *B. animalis CICC 24672* (ACS)

The supernatant of each sample (MRS, ACS, and BCS) was mixed with an extraction solution. After centrifugation, UHPLC-MS/MS analysis was performed. The UHPLC separation was processed using an EXIONLC System (Sciex), a SCIEX 6500 QTRAP plus triple quadrupole mass spectrometer (Sciex) equipped with an IonDrive Turbo V electrospray ionization interface. SCIEX Analyst Work Station_v1.6.3 and Sciex MultiQuant_v3.0.3 were employed for MRM data acquisition and processing.

### Statistical analysis

Statistical analysis was conducted with GraphPad Prism 9. Significant differences between groups were determined by a non-parametric test, unpaired Student's *t*-test, and one-way analysis of variance. Results were represented as mean ± SEM, and *P* < 0.05 was considered statistically significant, *P < 0.05, **P < 0.01, ***P < 0.001.

## Results

### *B. breve lw01* protects against colonic tumorigenesis in AOM/DSS-induced CRC mice

**Figure 1:**
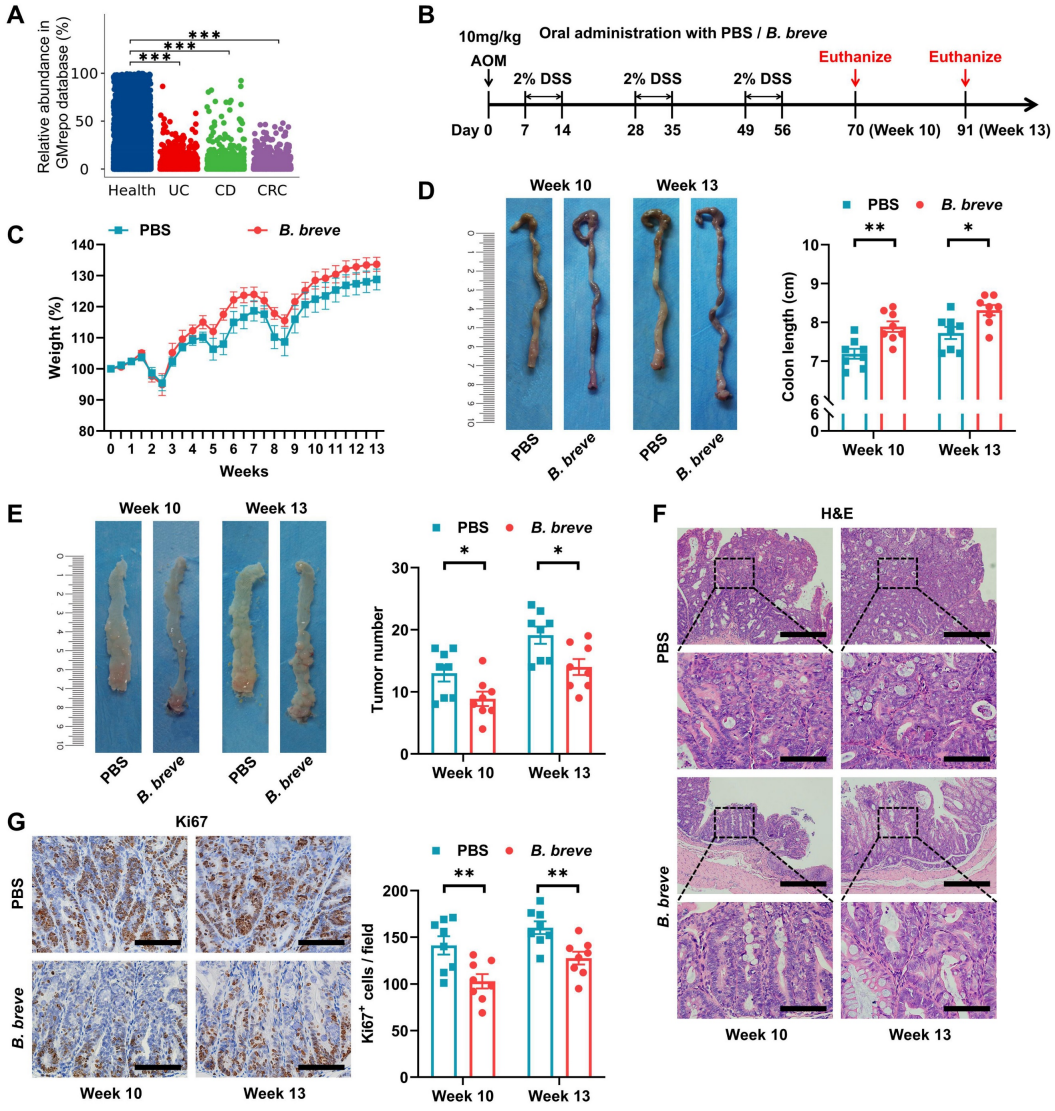
*B. breve lw01* protects against colonic tumorigenesis in AOM/DSS-induced CRC mice. (A) Fecal abundance of *Bifidobacterium* in healthy individuals, UC, CD, and CRC patients in the GMrepo database. (B) Schematic diagram for establishing CAC models induced by AOM/DSS in two groups (PBS or *B. breve lw01* was administered daily during CAC development). n=8 per group. (C) Weight changes relative to the initial weight during CAC development. (D) Representative colonic images and statistical histogram of colon length. (E) Representative colonic images and statistical histogram of tumor number. (F) Representative H&E staining for the distal colon of each group. Scale bars, 400 µm (upper), 100 µm (lower). (G) Representative IHC staining and quantitation of Ki67 in the distal colon. Scale bars, 100 µm. Data are represented as mean ± SEM. *P < 0.05, **P < 0.01, ***P < 0.001, ns: not significant. AOM: azoxymethane; *B. breve*: *Bifidobacterium breve*; CAC: colitis-associated colorectal cancer; CD: Crohn's disease; CRC: colorectal cancer; DSS: dextran sodium sulfate; UC: ulcerative colitis.

We first compared fecal *Bifidobacterium* levels in IBD and CRC patients with healthy individuals in the GMrepo database and detected a significant decrease in the abundance of *Bifidobacterium* in IBD and CRC patients (Figure 1A).

We previously reported the anti-tumor effect of a novel strain, *B. breve lw01* 20, which modulated intestinal immune cells 21. To further investigate its role in inhibiting colitis and delaying tumorigenesis, we treated a murine colitis-associated CRC model with *B. breve lw01* (Figure 1B). Although *B. breve lw01* failed to restore the reduction in body weight, it alleviated the colon shortening due to chronic colitis, compared with the PBS group (Figure 1C-D). More significantly, the treatment delayed tumor formation (Figure 1E and Figure S1A) and reduced histological injuries in the distal colon, reflected by the loss of histological structure and marked inflammatory cell infiltration at a low level, compared with the PBS group (Figure 1F). Furthermore, *B. breve lw01* decreased Ki67, p-STAT3, and activated-β-catenin expression in the tumor area (Figure 1G and Figure S1B), indicating that the bacteria could inhibit tumor cell proliferation and attenuate signaling pathways involved in tumorigenesis in CAC mice. No changes in body weight and colon length were found in normal mice following the gavage with *B. breve lw01* (Figure S2A-C). These results suggested that *B. breve lw01* ameliorates the intestinal inflammatory environment to abrogate carcinogenesis in AOM/DSS-induced CRC mice.

### *B. breve lw01* promotes differentiation from immature inflammatory macrophages to a homeostatic macrophage phenotype

**Figure 2:**
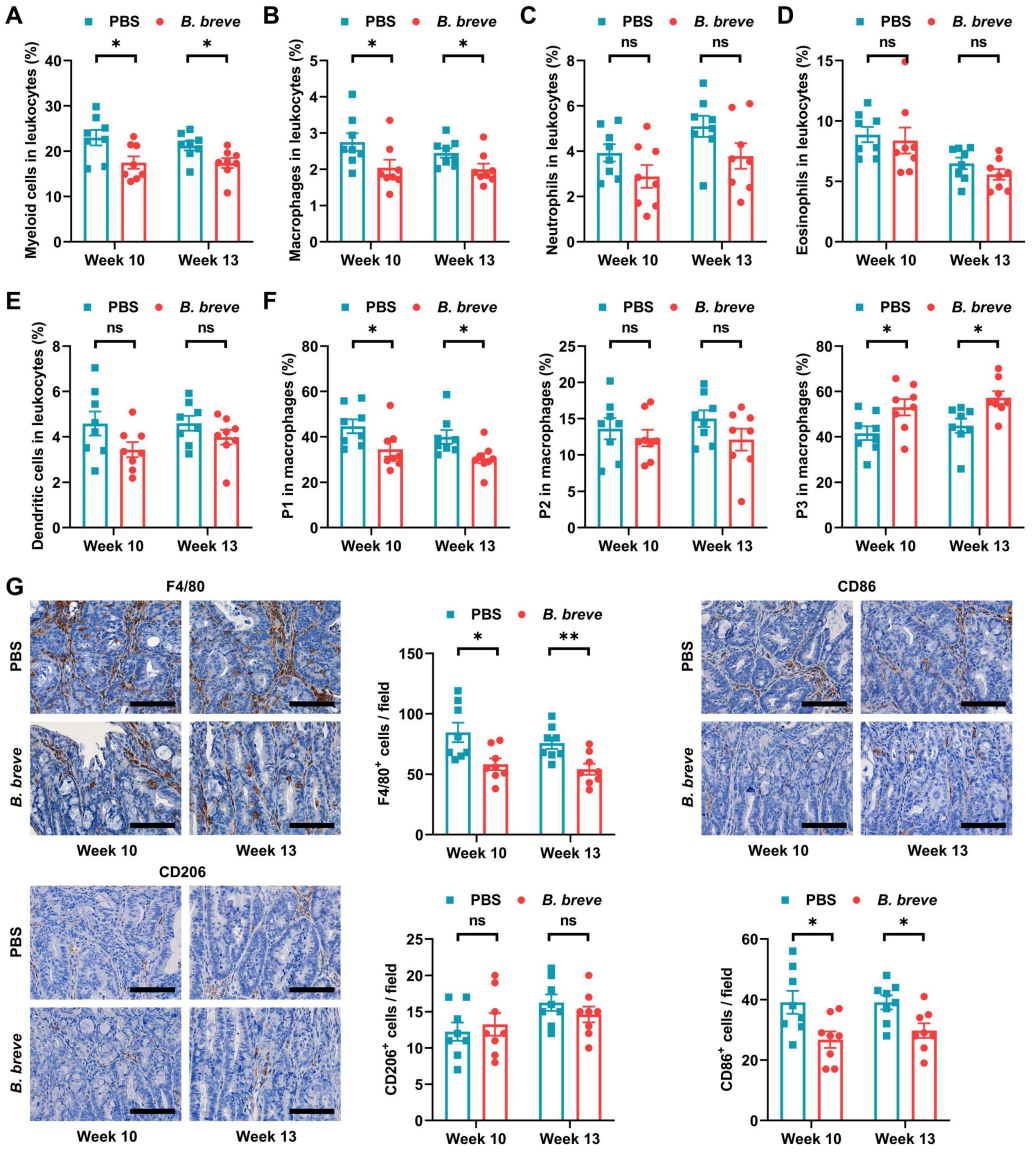
*B. breve lw01* promotes differentiation from immature inflammatory macrophages to a homeostatic macrophage phenotype. (A-F) Proportion of (A) myeloid cells, (B) macrophages, (C) neutrophils, (D) eosinophils, (E) dendritic cells, and (F) macrophage subsets in the colonic LP assessed by flow cytometry in CAC mice. (G) Representative IHC staining and quantitation of F4/80, CD86, and CD206 in the distal colon. Scale bars, 100 µm. Data are represented as mean ± SEM. *P < 0.05, **P < 0.01, ***P < 0.001, ns: not significant. *B. breve*: *Bifidobacterium breve*; CAC: colitis-associated colorectal cancer; LP: lamina propria; P1: immature colonic macrophage; P3: mature colonic macrophage.

Since infiltration of myeloid cells contributes to CAC carcinogenesis 27, we next investigated which myeloid cell subsets were targeted by *B. breve lw01* to reduce tumorigenesis. Flow cytometry was used to evaluate various myeloid cell subsets in colon tissues (Figure S3A). Supplementation with *B. breve lw01* decreased the proportion of colonized myeloid cells to reduce intestinal inflammation (Figure 2A). In addition, a prominent contraction of macrophages was noticed in the *B. breve* group compared with the PBS group (Figure 2B), while there was no difference in other immune cell subsets (neutrophils, eosinophils, and dendritic cells) (Figure 2C-E).

Next, we examined whether intestinal tumorigenesis could be due to the alteration in macrophage subpopulations induced by *B. breve lw01*. Three subsets of macrophages were assessed based on the expression of surface markers (Population 1 (P1), ICM: CD45^+^CD11b^+^F4/80^+^Ly6G^-^Siglec-F^-^Ly6C^hi^MHCII^-^, Population 2 (P2), CD45^+^CD11b^+^F4/80^+^Ly6G^-^Siglec-F^-^Ly6C^+^MHCII^+^, and Population 3 (P3), MCM: CD45^+^CD11b^+^F4/80^+^Ly6G^-^Siglec-F^-^Ly6C^-^MHCII^+^) 7, 10. *B. breve lw01* could upregulate MCMs and downregulate ICMs in CAC mice, while no significant changes were observed in P2 cells (Figure 2F), suggesting that the intestinal environment became stable.

Then we labeled pro-inflammatory and anti-inflammatory immune cells using CD86 and CD206, respectively. IHC analysis showed that *B. breve lw01* could debase CD86^+^ subsets to alleviate inflammation in CAC mice (Figure 2G). In addition, we analyzed the proportion of Foxp3^+^ or CD8^+^ T cells in the distal colon and found relatively low infiltration of both cell types (Figure S3B). We also evaluated the proportion of CD4^+^, CD4^+^CD25^+^, and CD8^+^ T cells in the MLN and spleen, and detected no significant changes (Figure S3C-E). Besides, the proportion of LP myeloid cells, MLN, and splenic T cells described above was similar in normal mice (Figure S4A-H). Collectively, a decrease in ICMs and an increase in MCMs were observed in the colonic LP, suggesting that *B. breve lw01* affects macrophage differentiation in CAC mice.

### Decreased tumorigenesis by *B. breve lw01* depends on macrophage participation

**Figure 3:**
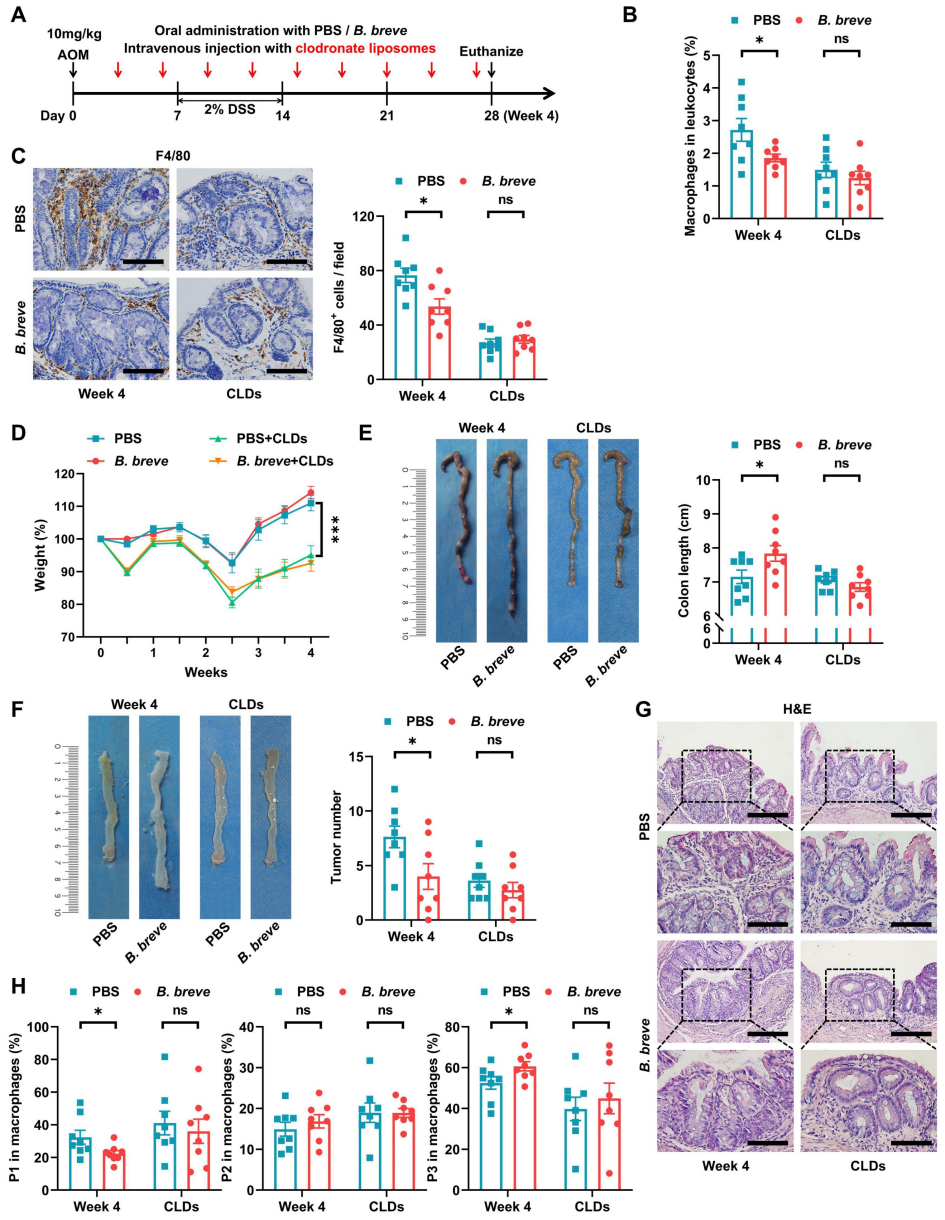
Decreased tumorigenesis by *B. breve lw01* depends on macrophage participation. (A) Schematic diagram for establishing macrophage-depleted models in four groups (PBS or *B. breve lw01* was administered daily, and CLDs were intravenously injected every three days during CAC development). n=8 per group. (B) Proportion of macrophages in the colonic LP assessed by flow cytometry in CAC mice. (C) Representative IHC staining and quantitation of F4/80 in the distal colon. Scale bars, 100 µm. (D) Weight changes relative to the initial weight during CAC development. (E) Representative colonic images and statistical histogram of colon length. (F) Representative colonic images and statistical histogram of tumor number. (G) Representative H&E staining for the distal colon of each group. Scale bars, 200 µm (upper), 100 µm (lower). (H) Proportion of macrophage subsets in the colonic LP assessed by flow cytometry in CAC mice. Data are represented as mean ± SEM. *P < 0.05, **P < 0.01, ***P < 0.001, ns: not significant. AOM: azoxymethane; *B. breve*: *Bifidobacterium breve*; CAC: colitis-associated colorectal cancer; CLD: clodronate liposome; DSS: dextran sodium sulfate; LP: lamina propria; P1: immature colonic macrophage; P3: mature colonic macrophage.

Subsequently, we assessed whether intestinal macrophages were involved in the protective role of *B. breve lw01* during CAC development. Considering the high lethality of CLDs, they were intravenously injected into mice through the lateral tail vein to accomplish macrophage depletion in the early CAC stage (Figure 3A). Flow cytometry and IHC analysis showed that CLDs significantly decreased the proportion of colonic macrophages (Figure 3B-C). Notably, macrophage ablation largely exacerbated body weight loss and abrogated the protective effect of *B. breve lw01*, which failed to improve the parameter associated with chronic colitis in CAC mice (Figure 3D-E). Furthermore, *B. breve lw01* could not reduce the number of tumors without the participation of macrophages (Figure 3F and Figure S5A). Histological examination further demonstrated that the beneficial effects of *B. breve lw01* largely diminished with the deletion of intestinal macrophages (Figure 3G).

Similarly, the ablation of macrophages annulled the alteration in the proportion of total and macrophage subsets induced by *B. breve lw01*; the remaining macrophages still expressed a similar proportion of immature phenotypes compared with the PBS+CLDs group (Figure 3B-C and 3H). Meanwhile, other myeloid cell subsets remained invariant between the two groups in the macrophage-depleted mice (Figure S5B-E). These results illustrated that the protective effects of *B. breve lw01* against CAC depend on colonic macrophages.

### *B. breve lw01* alters the transcriptional signature and the inflammatory response of colonic macrophages

**Figure 4:**
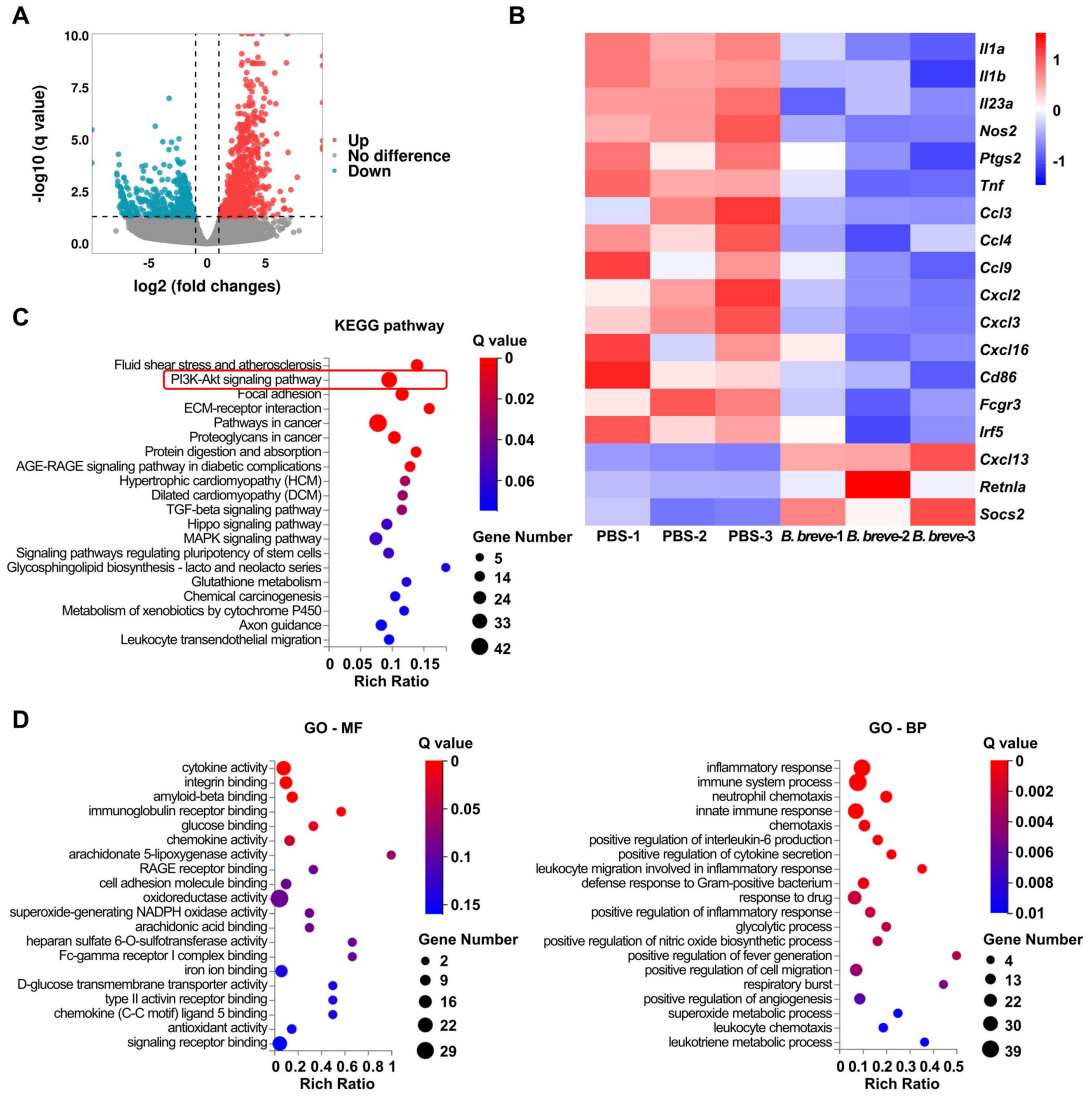
*B. breve lw01* alters the transcriptional signature and the inflammatory response of colonic macrophages. (A) Scatter plot showing DEGs of the total colonic LP macrophages in the *B. breve* group vs PBS group in CAC mice (Red dots represent upregulated DEGs, and cyan dots represent downregulated DEGs). (B) Heatmap showing DEGs related to inflammatory response, chemotaxis, and differentiation of colonic macrophages. (C) KEGG pathway enrichment analysis of upregulated DEGs (Dot size represents the number of DEGs, and dot color represents the corresponding *Q* value). (D) GO pathway enrichment analysis (molecular function and biological process) of downregulated DEGs (Dot size represents the number of DEGs, and dot color represents the corresponding *Q* value). *B. breve*: *Bifidobacterium breve*; CAC: colitis-associated colorectal cancer; DEG: differentially expressed gene; GO-BP: biological process of Gene Ontology; GO-MF: molecular function of Gene Ontology; KEGG: Kyoto Encyclopedia of Genes and Genomes; LP: lamina propria.

mRNA expression profiles of macrophages obtained from CAC mice distal colon using flow cytometry followed by RNA sequencing were analyzed to explore the role of *B. breve lw01* and the mechanisms promoting differentiation of macrophages. We observed differences in the expression profiles between the PBS and *B. breve* groups (Figure 4A) and focused on the genes involved in the inflammatory response, chemotaxis, and differentiation. Genes related to pro-inflammatory differentiation were significantly decreased in the *B. breve* group (Figure 4B). Kyoto Encyclopedia of Genes and Genomes (KEGG) pathway analysis revealed several distinct pathway patterns (Figure 4C). Moreover, Gene Ontology (GO) analysis of the downregulated genes identified significant inhibition of many inflammatory pathways in colonic macrophages, ranging from cytokine secretion to leukocyte chemotaxis (Figure 4D).

### *B. breve lw01* modulates gut microbiota of CAC mice and catabolizes L-tryptophan to release ILA

**Figure 5:**
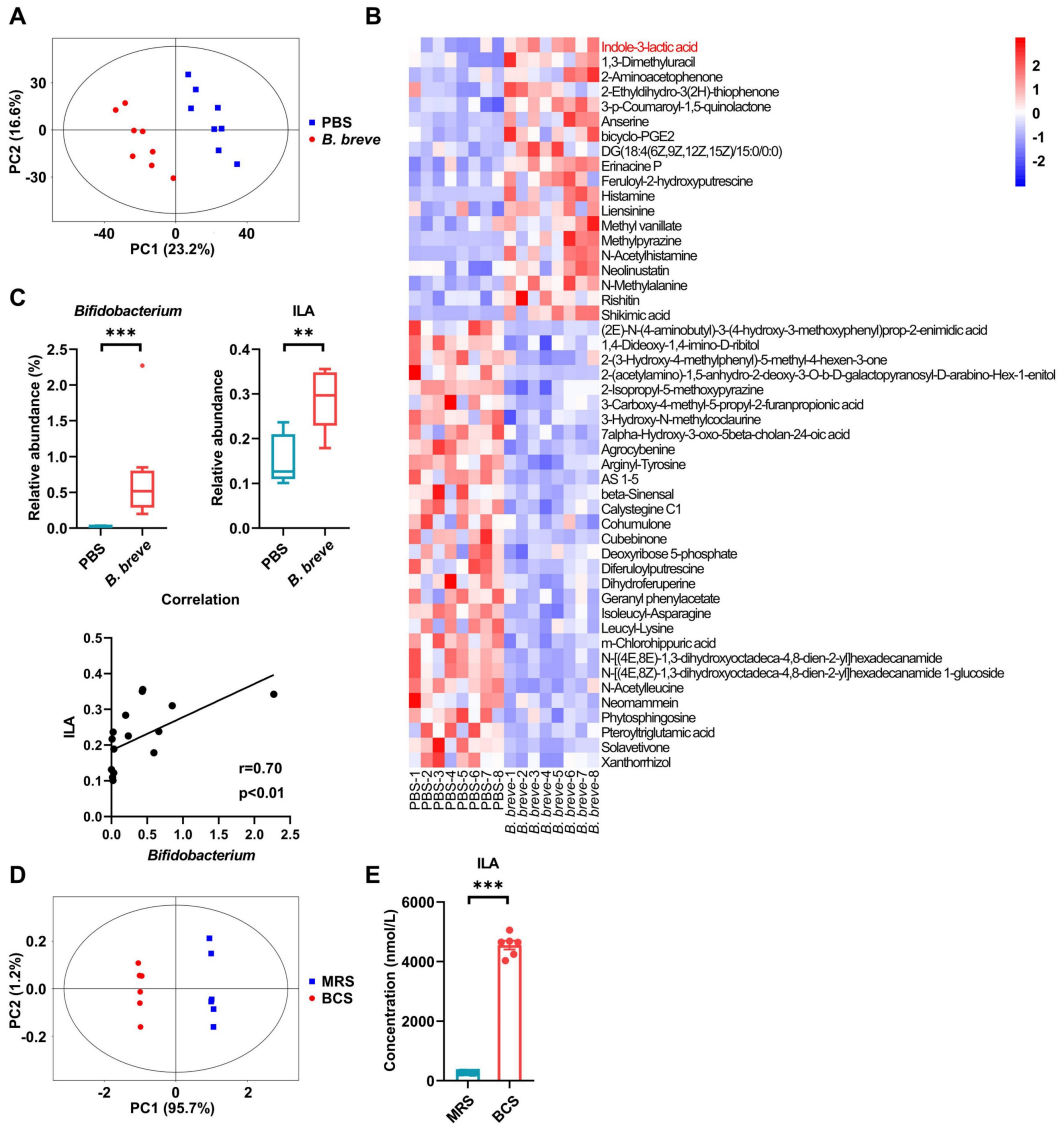
*B. breve lw01* catabolizes L-tryptophan to release ILA. (A) PCA plot of β-diversity to measure the composition of colonic microbial metabolites in the *B. breve* group vs PBS group in CAC mice. (B) Heatmap of differential colonic metabolites detected by LC-MS/MS. (C) Correlation analysis between *Bifidobacterium* and ILA in CAC mice. (D) PCA plot of β-diversity to measure L-tryptophan and its downstream metabolites between *B. breve lw01* and MRS supernatant. (E) Concentration for ILA production of *B. breve lw01* metabolizing L-tryptophan *in vitro*. Data are represented as mean ± SEM. *P < 0.05, **P < 0.01, ***P < 0.001, ns: not significant. *B. breve*: *Bifidobacterium breve*; BCS: the culture supernatant of *B. breve*; CAC: colitis-associated colorectal cancer; ILA: indole-3-lactic acid; LC-MS/MS: liquid chromatography-tandem mass spectrometry; MRS: de Man, Rogosa and Sharpe medium; PCA: principal component analysis.

We investigated the key functional components targeted by *B. breve lw01* by analyzing colonic contents using non-targeted LC-MS/MS after gavaging with *B. breve lw01* for 13 weeks. Principal component analysis (PCA) score plots showed separations between the PBS and *B. breve* groups (Figure 5A). Differential abundance analysis showed qualitative differences between the two groups (Figure 5B). Moreover, based on the correlation analysis, we found that ILA, a downstream metabolite of L-tryptophan, was positively correlated with *Bifidobacterium* (Figure 5C). To further confirm that *B. breve lw01* could convert L-tryptophan into ILA, we performed high throughput target detection of L-tryptophan and its downstream metabolites using the culture supernatant of *B. breve lw01*. The PCA plot of L-tryptophan and its downstream metabolites exhibited distinct separations between BCS and MRS (Figure 5D). Differential abundance analysis showed decreased L-tryptophan in BCS compared with the control culture supernatant (Figure S6A-B), suggesting that *B. breve lw01* could catabolize L-tryptophan. Also, ILA was highly enriched in BCS (Figure 5E and Figure S6A).

We further investigated the effects of *B. breve lw01* on gut microbiota by performing 16S rRNA gene sequencing on AOM/DSS mice colonic contents after gavaging with *B. breve lw01* for 13 weeks. The microbial richness indices showed no difference between the two groups (Figure S6C). Nevertheless, the colonic microbial community composition changed significantly after administering *B. breve lw01* (Figure S6D). In addition, *B. breve lw01* enriched probiotics and potentially depleted intestinal pathogens (Figure S6E-G). Moreover, some other genera that might produce ILA were also enriched in individual CAC mice following treatment with *B. breve lw01*. However, there was no statistically significant difference between the two groups, except for *Megamonas* (Figure S6H). In addition, a prominent amount of *Bifidobacterium* colonized in the colonic LP compared with the PBS group, which prompted the possibility that its derived ILA directly activated the colonic macrophages (Figure S6I). In brief, these results indicated that *B. breve lw01* can modulate gut microbiota and metabolize L-tryptophan to produce ILA.

### ILA alleviates LPS-induced pro-inflammatory response of murine BMDMs and macrophages derived from hPBMCs via the PI3K/AKT signaling pathway

**Figure 6:**
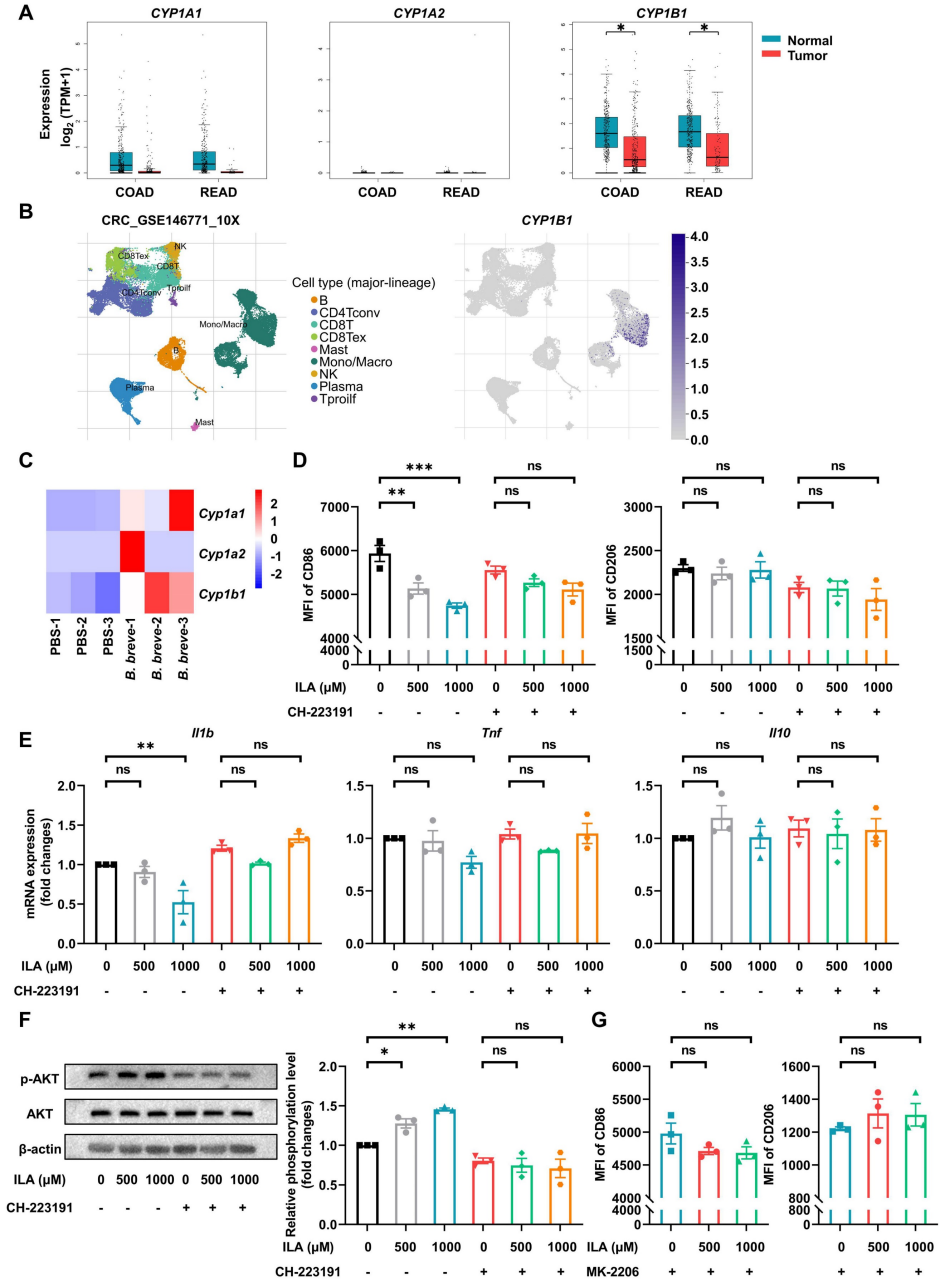
ILA alleviates LPS-induced pro-inflammatory response of murine BMDMs via the PI3K/AKT signaling pathway. (A) Gene expression of *CYP1A1*, *CYP1A2*, and *CYP1B1* in healthy individuals and CRC patients in the GEPIA database. (B) Gene expression of *CYP1B1* in different cell subsets of CRC patients in the TISCH database. (C) Gene expression of *Cyp1a1*, *Cyp1a2*, and *Cyp1b1* in total colonic LP macrophages of CAC mice between the two groups. (D) Effect of ILA with or without CH-223191 on the proportion of CD86 or CD206 in murine BMDMs, as tested by flow cytometry. (E) Gene expression of *Il1b*, *Tnf*, and *Il10* in murine BMDMs. (F) Representative Western blot images and statistical histogram of AKT phosphorylation levels in murine BMDMs. (G) Effect of ILA with or without MK-2206 on the proportion of CD86 or CD206 in murine BMDMs, as tested by flow cytometry. Data are represented as mean ± SEM. *P < 0.05, **P < 0.01, ***P < 0.001, ns: not significant. *B. breve*: *Bifidobacterium breve*; BMDM: bone marrow-derived macrophage; CAC: colitis-associated colorectal cancer; COAD: colon adenocarcinoma; CRC: colorectal cancer; ILA: indole-3-lactic acid; LP: lamina propria; LPS: lipopolysaccharide; MFI: mean fluorescence intensity; READ: rectum adenocarcinoma.

ILA has been reported to be a ligand of AhR 28, and activating AhR could regulate intestinal mucosal barrier integrity 29 and restrict inflammation 30. Also, AhR activation could drive macrophages to acquire a homeostatic phenotype 31. We analyzed the activation of AhR in CRC patients by the GEPIA database and found that the AhR-responsive gene *CYP1B1* was significantly decreased compared with healthy individuals (Figure 6A). The TISCH database analysis showed that *CYP1B1* was specifically expressed in monocytes/macrophages of CRC patients (Figure 6B). Besides, the AhR of intestinal macrophages was markedly activated in the *B. breve* group (Figure 6C). To investigate whether *B. breve lw01*-produced ILA could be responsible for macrophage differentiation in the anti-CAC effects, we verified its role in macrophage differentiation *in vitro*. We used CD86 and CD206 to mark the phenotypes of murine BMDMs and macrophages derived from hPBMCs. The pro-inflammatory differentiation of macrophages was disrupted by ILA (Figure 6D and Figure S7A). Adding ILA significantly decreased the mRNA expression of *Il1b*; however, using the AhR antagonist abolished the effects of ILA described above (Figure 6D-E and Figure S7A-B).

KEGG pathway enrichment analysis of the differentially expressed genes (DEGs) in colonic macrophages indicated that the upregulated genes were significantly enriched in regulating the PI3K/AKT signaling pathway (Figure 4C).

Previous studies showed that activation of the PI3K/AKT signaling pathway is essential in restricting the excessive immune response of macrophages and is considered a negative regulator of TLR signaling in macrophages 32, 33. We further validated the role of the PI3K/AKT signaling pathway in the phenotypic transformation of macrophages induced by ILA. Adding ILA increased AKT phosphorylation without changing total AKT protein levels, while co-treatment with the AhR antagonist CH-223191 reversed ILA-mediated AKT phosphorylation (Figure 6F and Figure S7C). We subsequently examined whether AKT signaling was involved in the ILA-mediated differentiation of macrophages. Flow cytometry analysis showed that pre-incubation of BMDMs and macrophages derived from hPBMCs with an AKT inhibitor MK-2206 abolished the phenotypic changes induced by ILA (Figure 6G and Figure S7D). Given that AKT activation could promote the development and progression of many tumors, we examined the effects of ILA on AKT phosphorylation in two murine CRC cell lines and found that ILA failed to promote AKT phosphorylation in CT26.WT and MC38 cells (Figure S7E). These results suggested that ILA induces phenotypic changes in macrophages and is associated with the reduction of inflammation by activating the PI3K/AKT signaling pathway.

### *B. breve lw01* releases ILA to protect against tumorigenesis by AhR-regulated macrophage differentiation

**Figure 7:**
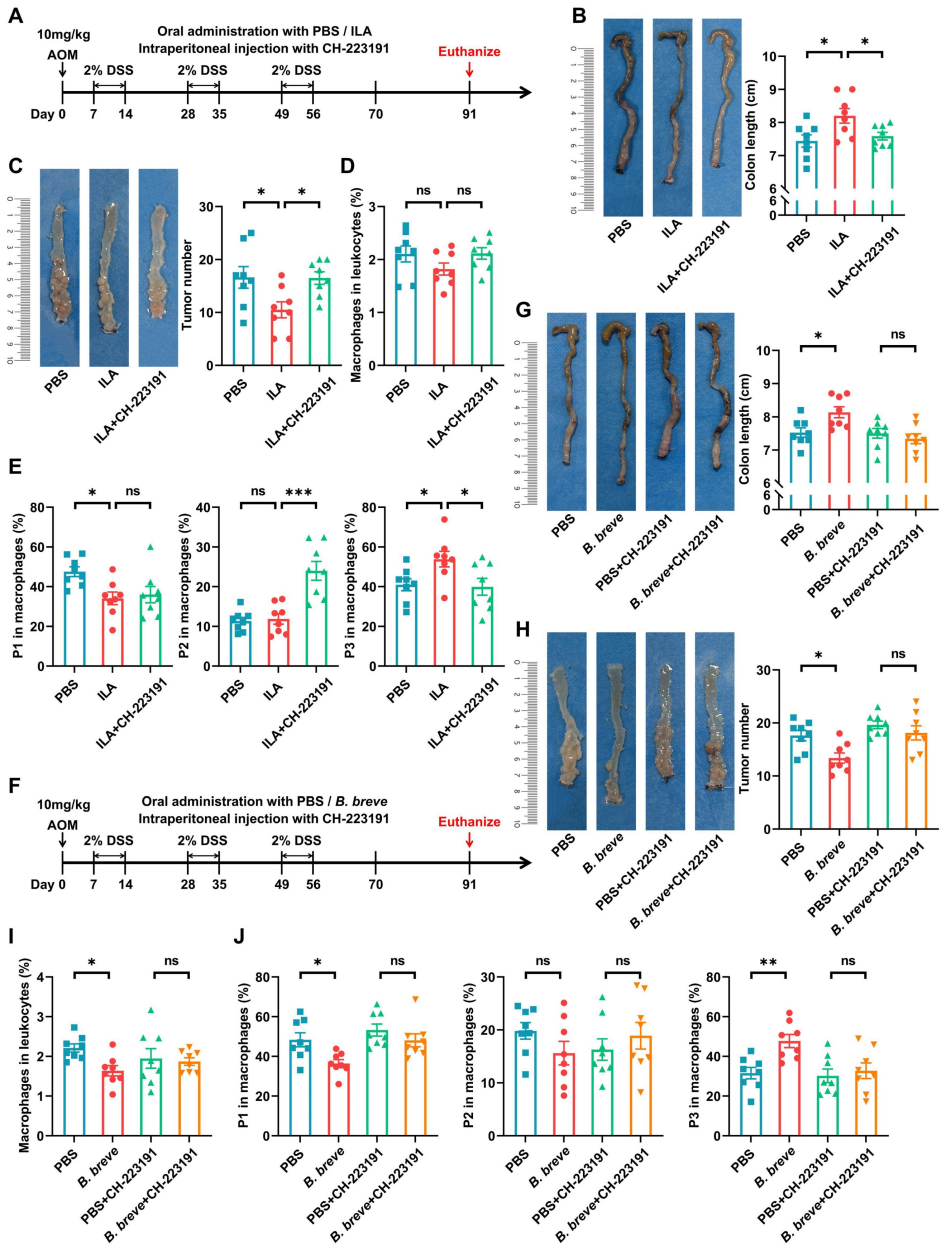
*B. breve lw01* releases ILA to protect against tumorigenesis by AhR-regulated macrophage differentiation. (A) Schematic diagram for establishing CAC models induced by AOM/DSS in three groups (PBS or ILA was administered daily, and CH-223191 was intraperitoneally injected every two days during CAC development). n=8 per group. (B) Representative colonic images and statistical histogram of colon length. (C) Representative colonic images and statistical histogram of tumor number. (D, E) Proportion of (D) macrophages and (E) macrophage subsets in the colonic LP assessed by flow cytometry in CAC mice. (F) Schematic diagram for establishing CAC models induced by AOM/DSS in four groups (PBS or *B. breve lw01* was administered daily, and CH-223191 was intraperitoneally injected every two days during CAC development). n=8 per group. (G) Representative colonic images and statistical histogram of colon length. (H) Representative colonic images and statistical histogram of tumor number. (I, J) Proportion of (I) macrophages and (J) macrophage subsets in the colonic LP assessed by flow cytometry in CAC mice. Data are represented as mean ± SEM. *P < 0.05, **P < 0.01, ***P < 0.001, ns: not significant. AhR: aryl hydrocarbon receptor; AOM: azoxymethane; *B. breve*: *Bifidobacterium breve*; CAC: colitis-associated colorectal cancer; DSS: dextran sodium sulfate; ILA: indole-3-lactic acid; LP: lamina propria; P1: immature colonic macrophage; P3: mature colonic macrophage.

To further confirm whether ILA could protect against intestinal tumorigenesis by regulating ICM maturation, CAC mice were treated with exogenous ILA (Figure 7A). Supplementation with ILA significantly reduced colon shortening (Figure 7B) and tumor number and size (Figure 7C and Figure S8A). Moreover, ILA reduced the proportion of ICMs and increased MCMs without changing the infiltration of total macrophages (Figure 7D-E); blocking the ILA receptor abolished the protective effects of ILA in CAC mice (Figure 7B-E and Figure S8A). Next, we labeled pro-inflammatory and anti-inflammatory immune cells with CD86 and CD206, respectively. IHC analysis revealed that ILA could debase CD86^+^ pro-inflammatory immune cells in CAC mice, and the ablation of AhR annulled the alteration of CD86^+^ subsets induced by ILA (Figure S8B).

Meanwhile, to further assess the contribution of ILA to *Bifidobacterium*-mediated protective effects, we used another strain, *B. animalis CICC 24672*, which could not release ILA gavaged to CAC mice for 4 weeks (Figure S8C-D). *B. animalis CICC 24672* failed to exert the anti-inflammation and anti-tumor effects in CAC mice (Figure S8E-F). Furthermore, *B. animalis CICC 24672* abolished the alteration in the proportion of total and macrophage subsets (Figure S8G-H).

Besides, we performed additional experiments to evaluate if antagonizing AhR could abolish *B. breve lw01*-mediated anti-inflammation and anti-tumor effects (Figure 7F). The AhR antagonist largely eliminated the protective effect of *B. breve lw01* in AOM/DSS-induced CRC mice, as evidenced by no significant difference in colon shortening (Figure 7G). Also, blocking AhR reversed *B. breve lw01*-induced tumor inhibition and MCMs upregulation (Figure 7H-J and Figure S8I). Collectively, these results suggested that ILA contributes to the tumor-suppressive effect of *B. breve lw01* via activating the macrophage AhR.

## Discussion

Coordinated interactions between the intestinal epithelium, microbiota, and host immune system maintain intestinal homeostasis 34. In this study, we found that *B. breve lw01* effectively reduced colitis-associated tumorigenesis by directing the maturation of immature inflammatory macrophages toward a homeostatic macrophage phenotype. We also discovered that ILA, a crucial metabolite generated by *B. breve lw01*, could be primarily involved in restoring intestinal homeostasis by activating AhR of macrophages. Although previous studies explored the effects of *Bifidobacterium* or ILA, verifying their association with anti-inflammation 15, 35, our present study elucidates, for the first time, that the differentiation of macrophages during colitis-associated tumorigenesis can be regulated by *Bifidobacterium* and its associated metabolites.

Chronic inflammation can accelerate the transformation of intestinal epithelial cells (IECs) into tumor cells 36. Our study demonstrated that *B. breve lw01* could attenuate colitis as evidenced by the reduced damage of histological structures and decreased infiltration of inflammatory cells, consistent with a previous study 37. Moreover, decreased inflammation could also downregulate signaling pathways associated with IEC tumorigenesis. These results suggested that *B. breve lw01* could prevent carcinogenesis by inhibiting inflammatory responses.

Macrophages have a critical function in tumor formation. In the early stage, excessive activation of immature macrophages promotes mutations and aggravates an inflammatory milieu suitable for tumorigenesis 38. Studies using preclinical models or human specimens confirmed inducing polarization of alternatively activated macrophages and/or inhibiting inflammatory signaling pathways in clinical IBD therapy and prevention of tumorigenesis 5. Also, anti-TNF treatment or immunosuppressive therapy can inhibit the incidence rate of IBD-associated CRC 2, 3. Other studies demonstrated that ICMs are the predominant source of IL1β and TNFα in CAC mice and key to transforming cells during the tumor initiation stage 10, 39. In this study, we observed that supplementation with *B. breve lw01* could modulate the quantity and composition of colonic macrophages, inducing a homeostatic state and delay tumorigenesis.

However, a moderate method to regulate the quantity and quality of macrophages is needed. Excessive macrophage depletion results in a devastating blow to the immune function 40. Although there was tumor inhibition in macrophage-depleted mice, the severity of weight loss exacerbated their mortality. The decrease in tumors using CLDs could result in inadequate restoration of the damaged intestinal epithelium, consequently reducing the possibility of mismatch repair partially 39, 41. However, gut microbiota could constantly stimulate the colonic macrophages due to the absence of intestinal epithelial barrier, resulting in increased inflammatory cell accumulation and aggravating intestinal inflammation. Long-term administration of *B. breve lw01* could achieve a balanced distribution of macrophage subsets. Our concept of selectively blocking excrescent pro-inflammatory macrophage subset accumulation without affecting the differentiation of homeostatic macrophages to reduce intestinal epithelium damage may be a promising strategy to prevent CAC.

Some studies suggested that the gut microbiota variation may be related to tumor initiation 42, 43. We found that *B. breve lw01* could enrich the abundance of some probiotics while decreasing potential pathogenic species. Our results suggested that probiotics like *B. breve lw01* might protect against intestinal tumorigenesis by modulating gut microbial composition. Although eliminating all gut microbiota could lead to tumor prevention in CAC mice 44, strong epidemiological evidence links the gut microbiota disruption by early antibiotic exposure to IBD in people 45, 46. Furthermore, continuous dysregulation of intestinal immune cells induced by antibiotics may lead to other serious diseases 47. Using probiotics or other treatments to maintain the potentially pathogenic species in low abundance may become a satisfactory method to relieve CAC. This study mainly focused on the role of metabolites produced by specific bacterial strains on CAC development. In the future, we plan to assess the changes in the microbial community affected by *B. breve lw01* on CAC development.

Gut microbiota-derived metabolites mediate many biological effects. The primary metabolites of *Bifidobacterium* comprise short-chain fatty acids and tryptophan catabolites 22, 48. L-tryptophan, an essential aromatic amino acid and its downstream derivatives are considered vital in the host-gut microbiota interactions by activating AhR in immune cells 49, 50. As one of L-tryptophan catabolites, ILA was enriched in both *B. breve lw01* culture supernatant and colonic contents of *B. breve lw01*-treated AOM/DSS mice. In contrast to adult- or animal-associated *Bifidobacterium* species, *B. breve* isolated from the feces of infants could process relatively higher ILA levels, providing a colonial advantage in the infant's gut to relieve intestinal inflammation 51, 52.

The impact of various strains on a specific disease differs significantly 53. *B. animalis CICC 24672* isolated from chicken manure failed to replicate the observed protective effects. Another study showed that the adult-type *Bifidobacterium adolescentis* might offer distinct ways to inhibit tumor growth 54. We focused more on a novel strain *B. breve lw01* isolated from infants and detected its potentially preventive effects on tumorigenesis. Recent studies revealed that ILA produced by *Lactobacillus* can affect the Th17 or CD8^+^ T cell functions 55, 56. Our findings provided a new insight into *Bifidobacterium*-mediated intestinal tumorigenesis inhibition via macrophages. Furthermore, our study showed that ILA could disrupt pro-inflammatory macrophage differentiation *in vitro*, as the addition of ILA markedly decreased the expression of *Il1b* and activated AKT phosphorylation, which is crucial for restraining macrophage immune responses 32, 33. The tumor-suppressive effect of *B. breve lw01* by activating AhR could be ascribed to its protective catabolites. The ablation of AhR could reverse ILA-induced MCM upregulation. While P2 macrophages were upregulated significantly in the ILA+CH-223191 group, their differentiation was compromised by AhR ablation. In the future, we plan to analyze the gene(s) responsible for generating ILA 35 in *B. breve lw01*.

Collectively, our findings established the link between *B. breve lw01* and its metabolite-ILA and macrophage differentiation in colitis-associated tumorigenesis. *B. breve lw01* holds promise as a therapeutic agent for mitigating inflammation-associated tumorigenesis by reshaping the immune landscape. Our study provides a rational and mechanistic basis for novel probiotic therapeutic strategies to determine the clinically protective effects of microbial AhR ligands, like ILA, for colitis and CAC patients and is expected to inspire further investigations.

## Supplementary Material

Supplementary figures and table.

## Abbreviations

ACS: the culture supernatant of *B. animalis*
AhR: aryl hydrocarbon receptor
AOM: azoxymethane
ASV: amplicon sequence variant
*B. animalis*: *Bifidobacterium animalis*
*B. breve*: *Bifidobacterium breve*
BCS: the culture supernatant of *B. breve*
BMDM: bone marrow-derived macrophage
CAC: colitis-associated colorectal cancer
CD: Crohn's disease
CFU: colony-forming unit
CLD: clodronate liposome
CRC: colorectal cancer
DAPI: 4',6-diamidino-2-phenylindole
DEG: differentially expressed gene
DMEM: Dulbecco's modified Eagle medium
DNB: DNA nanoball
DSS: dextran sodium sulfate
FBS: fetal bovine serum
FISH: fluorescence *in situ* hybridization
GEPIA: Gene Expression Profiling Interactive Analysis
GO: Gene Ontology
H&E: hematoxylin and eosin
hPBMC: human peripheral blood mononuclear cell
IBD: inflammatory bowel disease
ICM: immature colonic macrophage
IEC: intestinal epithelial cell
IHC: immunohistochemistry
ILA: indole-3-lactic acid
KEGG: Kyoto Encyclopedia of Genes and Genomes
LC-MS/MS: liquid chromatography-tandem mass spectrometry
LP: lamina propria
LPS: lipopolysaccharide
MCM: mature colonic macrophage
MLN: mesenteric lymph node
MRS: de Man, Rogosa and Sharpe
OTU: operational taxonomic unit
PCA: principal component analysis
PCoA: principal coordinate analysis
qRT-PCR: quantitative real-time PCR
RPMI-1640: Roswell Park Memorial Institute-1640
SPF: specific pathogen-free
TISCH: Tumor Immune Single-cell Hub
UC: ulcerative colitis
UHPLC: ultra-high performance liquid chromatography

## References

[1] Shah SC, Itzkowitz SH (2022). Colorectal Cancer in Inflammatory Bowel Disease: Mechanisms and Management. Gastroenterology.

[2] Baars JE, Looman CW, Steyerberg EW, Beukers R, Tan AC, Weusten BL (2011). The risk of inflammatory bowel disease-related colorectal carcinoma is limited: results from a nationwide nested case-control study. Am J Gastroenterol.

[3] Lu MJ, Qiu XY, Mao XQ, Li XT, Zhang HJ (2018). Systematic review with meta-analysis: thiopurines decrease the risk of colorectal neoplasia in patients with inflammatory bowel disease. Aliment Pharmacol Ther.

[4] Delfini M, Stakenborg N, Viola MF, Boeckxstaens G (2022). Macrophages in the gut: Masters in multitasking. Immunity.

[5] Na YR, Stakenborg M, Seok SH, Matteoli G (2019). Macrophages in intestinal inflammation and resolution: a potential therapeutic target in IBD. Nat Rev Gastroenterol Hepatol.

[6] Locati M, Curtale G, Mantovani A (2020). Diversity, Mechanisms, and Significance of Macrophage Plasticity. Annu Rev Pathol.

[7] Bain CC, Bravo-Blas A, Scott CL, Perdiguero EG, Geissmann F, Henri S (2014). Constant replenishment from circulating monocytes maintains the macrophage pool in the intestine of adult mice. Nat Immunol.

[8] Bain CC, Scott CL, Uronen-Hansson H, Gudjonsson S, Jansson O, Grip O (2013). Resident and pro-inflammatory macrophages in the colon represent alternative context-dependent fates of the same Ly6Chi monocyte precursors. Mucosal Immunol.

[9] Rivollier A, He J, Kole A, Valatas V, Kelsall BL (2012). Inflammation switches the differentiation program of Ly6Chi monocytes from antiinflammatory macrophages to inflammatory dendritic cells in the colon. J Exp Med.

[10] Yang Y, Li L, Xu C, Wang Y, Wang Z, Chen M (2020). Cross-talk between the gut microbiota and monocyte-like macrophages mediates an inflammatory response to promote colitis-associated tumourigenesis. Gut.

[11] Shao X, Sun S, Zhou Y, Wang H, Yu Y, Hu T (2021). Bacteroides fragilis restricts colitis-associated cancer via negative regulation of the NLRP3 axis. Cancer Lett.

[12] Yang W, Cong Y (2021). Gut microbiota-derived metabolites in the regulation of host immune responses and immune-related inflammatory diseases. Cell Mol Immunol.

[13] Rooks MG, Garrett WS (2016). Gut microbiota, metabolites and host immunity. Nat Rev Immunol.

[14] Ansaldo E, Slayden LC, Ching KL, Koch MA, Wolf NK, Plichta DR (2019). Akkermansia muciniphila induces intestinal adaptive immune responses during homeostasis. Science.

[15] Henrick BM, Rodriguez L, Lakshmikanth T, Pou C, Henckel E, Arzoomand A (2021). Bifidobacteria-mediated immune system imprinting early in life. Cell.

[16] Cervantes-Barragan L, Chai JN, Tianero MD, Di Luccia B, Ahern PP, Merriman J (2017). Lactobacillus reuteri induces gut intraepithelial CD4(+)CD8αα(+) T cells. Science.

[17] Sivan A, Corrales L, Hubert N, Williams JB, Aquino-Michaels K, Earley ZM (2015). Commensal Bifidobacterium promotes antitumor immunity and facilitates anti-PD-L1 efficacy. Science.

[18] Liu A, Ma T, Xu N, Jin H, Zhao F, Kwok LY (2021). Adjunctive Probiotics Alleviates Asthmatic Symptoms via Modulating the Gut Microbiome and Serum Metabolome. Microbiol Spectr.

[19] Groeger D, O'Mahony L, Murphy EF, Bourke JF, Dinan TG, Kiely B (2013). Bifidobacterium infantis 35624 modulates host inflammatory processes beyond the gut. Gut Microbes.

[20] Wang L, Wang Y, Li Q, Tian K, Xu L, Liu G (2019). Exopolysaccharide, Isolated From a Novel Strain Bifidobacterium breve lw01 Possess an Anticancer Effect on Head and Neck Cancer - Genetic and Biochemical Evidences. Front Microbiol.

[21] Li Q, Li Y, Wang Y, Xu L, Guo Y, Wang Y (2021). Oral administration of Bifidobacterium breve promotes antitumor efficacy via dendritic cells-derived interleukin 12. Oncoimmunology.

[22] Fang Z, Pan T, Li L, Wang H, Zhu J, Zhang H (2022). Bifidobacterium longum mediated tryptophan metabolism to improve atopic dermatitis via the gut-skin axis. Gut Microbes.

[23] Neufert C, Becker C, Neurath MF (2007). An inducible mouse model of colon carcinogenesis for the analysis of sporadic and inflammation-driven tumor progression. Nat Protoc.

[24] Wu S, Sun C, Li Y, Wang T, Jia L, Lai S (2020). GMrepo: a database of curated and consistently annotated human gut metagenomes. Nucleic Acids Res.

[25] Tang Z, Li C, Kang B, Gao G, Li C, Zhang Z (2017). GEPIA: a web server for cancer and normal gene expression profiling and interactive analyses. Nucleic Acids Res.

[26] Sun D, Wang J, Han Y, Dong X, Ge J, Zheng R (2021). TISCH: a comprehensive web resource enabling interactive single-cell transcriptome visualization of tumor microenvironment. Nucleic Acids Res.

[27] Katoh H, Wang D, Daikoku T, Sun H, Dey SK, Dubois RN (2013). CXCR2-expressing myeloid-derived suppressor cells are essential to promote colitis-associated tumorigenesis. Cancer Cell.

[28] Vrzalová A, Pečinková P, Illés P, Gurská S, Džubák P, Szotkowski M (2022). Mixture Effects of Tryptophan Intestinal Microbial Metabolites on Aryl Hydrocarbon Receptor Activity. Int J Mol Sci.

[29] Scott SA, Fu J, Chang PV (2020). Microbial tryptophan metabolites regulate gut barrier function via the aryl hydrocarbon receptor. Proc Natl Acad Sci U S A.

[30] Domínguez-Acosta O, Vega L, Estrada-Muñiz E, Rodríguez MS, Gonzalez FJ, Elizondo G (2018). Activation of aryl hydrocarbon receptor regulates the LPS/IFNγ-induced inflammatory response by inducing ubiquitin-proteosomal and lysosomal degradation of RelA/p65. Biochem Pharmacol.

[31] Shinde R, Hezaveh K, Halaby MJ, Kloetgen A, Chakravarthy A, da Silva Medina T (2018). Apoptotic cell-induced AhR activity is required for immunological tolerance and suppression of systemic lupus erythematosus in mice and humans. Nat Immunol.

[32] Luyendyk JP, Schabbauer GA, Tencati M, Holscher T, Pawlinski R, Mackman N (2008). Genetic analysis of the role of the PI3K-Akt pathway in lipopolysaccharide-induced cytokine and tissue factor gene expression in monocytes/macrophages. J Immunol.

[33] Covarrubias AJ, Aksoylar HI, Yu J, Snyder NW, Worth AJ, Iyer SS (2016). Akt-mTORC1 signaling regulates Acly to integrate metabolic input to control of macrophage activation. Elife.

[34] Belkaid Y, Harrison OJ (2017). Homeostatic Immunity and the Microbiota. Immunity.

[35] Laursen MF, Sakanaka M, von Burg N, Mörbe U, Andersen D, Moll JM (2021). Bifidobacterium species associated with breastfeeding produce aromatic lactic acids in the infant gut. Nat Microbiol.

[36] Greten FR, Grivennikov SI (2019). Inflammation and Cancer: Triggers, Mechanisms, and Consequences. Immunity.

[37] Chen Y, Yang B, Stanton C, Ross RP, Zhao J, Zhang H (2021). Bifidobacterium pseudocatenulatum Ameliorates DSS-Induced Colitis by Maintaining Intestinal Mechanical Barrier, Blocking Proinflammatory Cytokines, Inhibiting TLR4/NF-κB Signaling, and Altering Gut Microbiota. J Agric Food Chem.

[38] Cendrowicz E, Sas Z, Bremer E, Rygiel TP (2021). The Role of Macrophages in Cancer Development and Therapy. Cancers (Basel).

[39] Shin AE, Tesfagiorgis Y, Larsen F, Derouet M, Zeng PYF, Good HJ (2023). F4/80(+)Ly6C(high) Macrophages Lead to Cell Plasticity and Cancer Initiation in Colitis. Gastroenterology.

[40] Lee B, Qiao L, Kinney B, Feng GS, Shao J (2014). Macrophage depletion disrupts immune balance and energy homeostasis. PLoS One.

[41] Bader JE, Enos RT, Velázquez KT, Carson MS, Nagarkatti M, Nagarkatti PS (2018). Macrophage depletion using clodronate liposomes decreases tumorigenesis and alters gut microbiota in the AOM/DSS mouse model of colon cancer. Am J Physiol Gastrointest Liver Physiol.

[42] Chung Y, Ryu Y, An BC, Yoon YS, Choi O, Kim TY (2021). A synthetic probiotic engineered for colorectal cancer therapy modulates gut microbiota. Microbiome.

[43] Wang Z, Hua W, Li C, Chang H, Liu R, Ni Y (2019). Protective Role of Fecal Microbiota Transplantation on Colitis and Colitis-Associated Colon Cancer in Mice Is Associated With Treg Cells. Front Microbiol.

[44] Zackular JP, Baxter NT, Iverson KD, Sadler WD, Petrosino JF, Chen GY (2013). The gut microbiome modulates colon tumorigenesis. mBio.

[45] Kronman MP, Zaoutis TE, Haynes K, Feng R, Coffin SE (2012). Antibiotic exposure and IBD development among children: a population-based cohort study. Pediatrics.

[46] Shaw SY, Blanchard JF, Bernstein CN (2010). Association between the use of antibiotics in the first year of life and pediatric inflammatory bowel disease. Am J Gastroenterol.

[47] Scott NA, Andrusaite A, Andersen P, Lawson M, Alcon-Giner C, Leclaire C (2018). Antibiotics induce sustained dysregulation of intestinal T cell immunity by perturbing macrophage homeostasis. Sci Transl Med.

[48] Song Q, Zhang X, Liu W, Wei H, Liang W, Zhou Y (2023). Bifidobacterium pseudolongum-generated acetate suppresses non-alcoholic fatty liver disease-associated hepatocellular carcinoma. J Hepatol.

[49] Roager HM, Licht TR (2018). Microbial tryptophan catabolites in health and disease. Nat Commun.

[50] Shinde R, McGaha TL (2018). The Aryl Hydrocarbon Receptor: Connecting Immunity to the Microenvironment. Trends Immunol.

[51] Sakurai T, Odamaki T, Xiao JZ (2019). Production of Indole-3-Lactic Acid by Bifidobacterium Strains Isolated fromHuman Infants. Microorganisms.

[52] Huang W, Cho KY, Meng D, Walker WA (2021). The impact of indole-3-lactic acid on immature intestinal innate immunity and development: a transcriptomic analysis. Sci Rep.

[53] Wang L, Chai M, Wang J, Yu Q, Wang G, Zhang H (2022). Bifidobacterium longum relieves constipation by regulating the intestinal barrier of mice. Food Funct.

[54] Lin Y, Fan L, Qi Y, Xu C, Jia D, Jiang Y (2023). Bifidobacterium adolescentis induces Decorin(+) macrophages via TLR2 to suppress colorectal carcinogenesis. J Exp Clin Cancer Res.

[55] Han JX, Tao ZH, Wang JL, Zhang L, Yu CY, Kang ZR (2023). Microbiota-derived tryptophan catabolites mediate the chemopreventive effects of statins on colorectal cancer. Nat Microbiol.

[56] Zhang Q, Zhao Q, Li T, Lu L, Wang F, Zhang H (2023). Lactobacillus plantarum-derived indole-3-lactic acid ameliorates colorectal tumorigenesis via epigenetic regulation of CD8(+) T cell immunity. Cell Metab.

