# Supplementary material for: Bifidobacterium breve-derived indole-3-lactic acid ameliorates colitis-associated tumorigenesis by directing the differentiation of immature colonic macrophages
Source: Theranostics. 2024 Apr 22;14(7):2719–35. doi: 10.7150/thno.92350 (PMC11103503; doi:10.7150/thno.92350)
Supplement: Supplementary file 1 — Supplementary figures and table. [file thnov14p2719s1.pdf]

Supplementary tables

Table S1: Antibodies

Antibodies	Use	Source	Identifier	Clone
CD45-FITC	Flow cytometry	BioLegend	103108	30-F11
CD11b-BV605	Flow cytometry	BioLegend	101257	M1/70
F4/80-APC/Cy7	Flow cytometry	BioLegend	123118	BM8
Ly6G-BV650	Flow cytometry	BioLegend	127641	1A8
Siglec-F-PE	Flow cytometry	BioLegend	155506	S17007L
CD11c-PE/Cy7	Flow cytometry	BioLegend	117318	N418
MHCII-APC	Flow cytometry	BioLegend	107614	M5/114.15.2
Ly6C-BV421	Flow cytometry	BioLegend	128032	HK1.4
7-AAD	Flow cytometry	BioLegend	420404	-
CD45-BV510	Flow cytometry	BioLegend	103138	30-F11
CD3-APC/Cy7	Flow cytometry	BioLegend	100222	17A2
CD4-FITC	Flow cytometry	BioLegend	100510	RM4-5
CD25-PE	Flow cytometry	BioLegend	102008	PC61
CD8α-APC	Flow cytometry	BioLegend	100712	53-6.7
CD11b-APC	Flow cytometry	BioLegend	101212	M1/70
F4/80-BV421	Flow cytometry	BioLegend	123132	BM8
CD86-PE/Cy7	Flow cytometry	BioLegend	105014	GL-1
CD206-PE	Flow cytometry	BioLegend	141706	C068C2
TruStain FcX (mouse)	Flow cytometry	BioLegend	156604	S17011E
CD68-BV421	Flow cytometry	BioLegend	333828	Y1/82A
CD86-PE/Cy7	Flow cytometry	BioLegend	305422	IT2.2
CD206-PE	Flow cytometry	BioLegend	321106	15-2
TruStain FcX (human)	Flow cytometry	BioLegend	422302	-
Ki67	Immunohistochemistry	Cell Signaling Technology	12202	D3B5
p-STAT3	Immunohistochemistry	Cell Signaling Technology	9145	D3A7
Activated-β-catenin	Immunohistochemistry	Cell Signaling Technology	19807	D2U8Y

F4/80	Immunohistochemistry	Cell Signaling Technology	30325	D4C8V
CD86	Immunohistochemistry	Cell Signaling Technology	19589	E5W6H
CD206	Immunohistochemistry	Cell Signaling Technology	24595	E6T5J
Foxp3	Immunohistochemistry	Cell Signaling Technology	12653	D6O8R
CD8α	Immunohistochemistry	Cell Signaling Technology	98941	D4W2Z
p-AKT	Western blotting	Cell Signaling Technology	4060	D9E
AKT (Pan)	Western blotting	Cell Signaling Technology	4691	C67E7
β-actin	Western blotting	ZSGB-BIO	TA-09	OTI1

Table S2: Specific primer sequences

Gene name	Forward	Reverse
Mus-Il1b	TTCAGGCAGGCAGTATCACTC	GAAGGTCCACGGGAAAGACAC
Mus-Il10	GCTGGACAACATACTGCTAACC	ATTTCCGATAAGGCTTGGCAA
Mus-Tnf	CAGGCGGTGCCTATGTCTC	CGATCACCCCGAAGTTCAGTAG
Mus-Actb	GTGACGTTGACATCCGTAAAGA	GCCGGACTCATCGTACTCC
Homo-IL1B	AGCTACGAATCTCCGACCAC	CGTTATCCCATGTGTCGAAGAA
Homo-IL10	TCAAGGCGCATGTGAACTCC	GATGTCAAACTCACTCATGGCT
Homo-TNF	CCTCTCTCTAATCAGCCCTCTG	GAGGACCTGGGAGTAGATGAG
Homo-ACTB	AGAAAATCTGGCACCACACC	TAGCACAGCCTGGATAGCAA

Supplementary figures

Figure S1

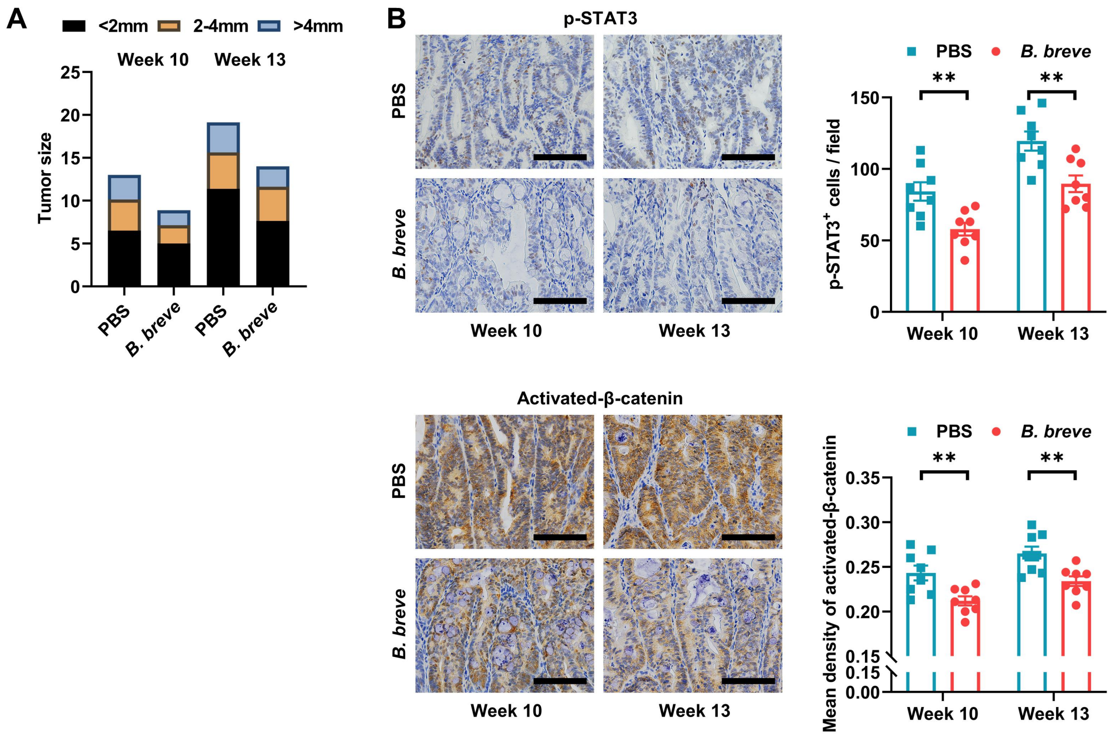

Figure S1: Supplementation with *B. breve lw01* alleviates tumorigenesis induced by AOM/DSS. (A) Statistical histogram of tumor size in CAC mice. (B) Representative IHC staining and quantitation of p-STAT3 and activated-β-catenin in the distal colon. Scale bars, 100 μm. Data are represented as mean ± SEM. **P* < 0.05, ***P* < 0.01, ****P* < 0.001, ns: not significant. AOM: azoxymethane; *B. breve*: *Bifidobacterium breve*; CAC: colitis-associated colorectal cancer; DSS: dextran sodium sulfate.

Figure S2

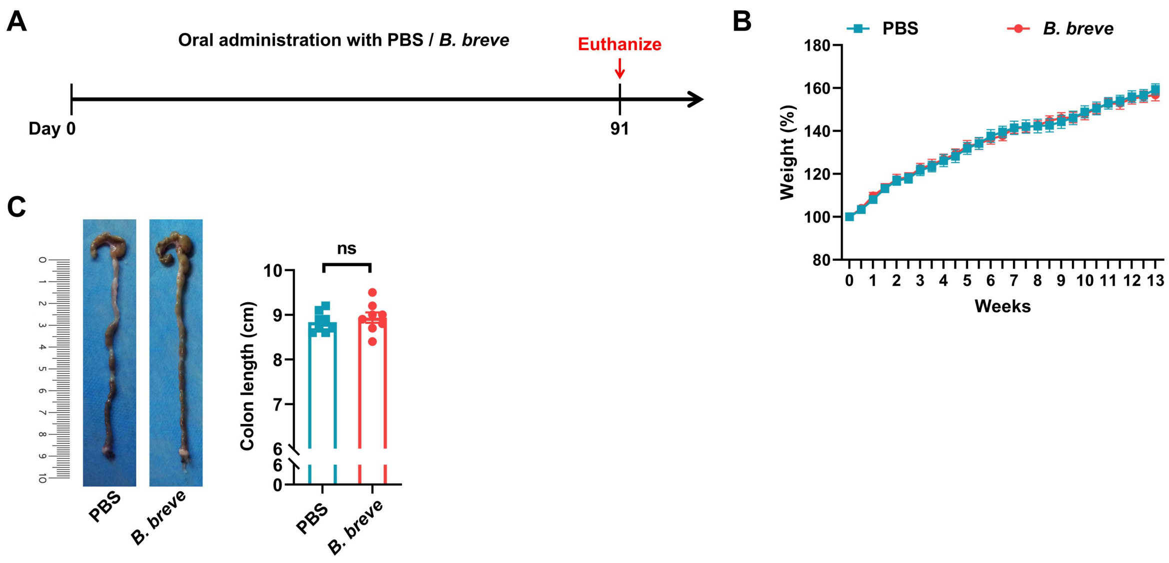

Figure S2: Clinical symptoms are not changed in normal mice. (A) Schematic diagram of two groups (PBS or *B. breve lw01* was administered daily during the entire period without other treatments). n=8 per group. (B) Weight changes relative to the initial weight during the whole stage. (C) Representative colonic images and statistical histogram of colon length. Data are represented as mean ± SEM. **P* < 0.05, ***P* < 0.01, ****P* < 0.001, ns: not significant. *B. breve*: *Bifidobacterium breve*.

Figure S3

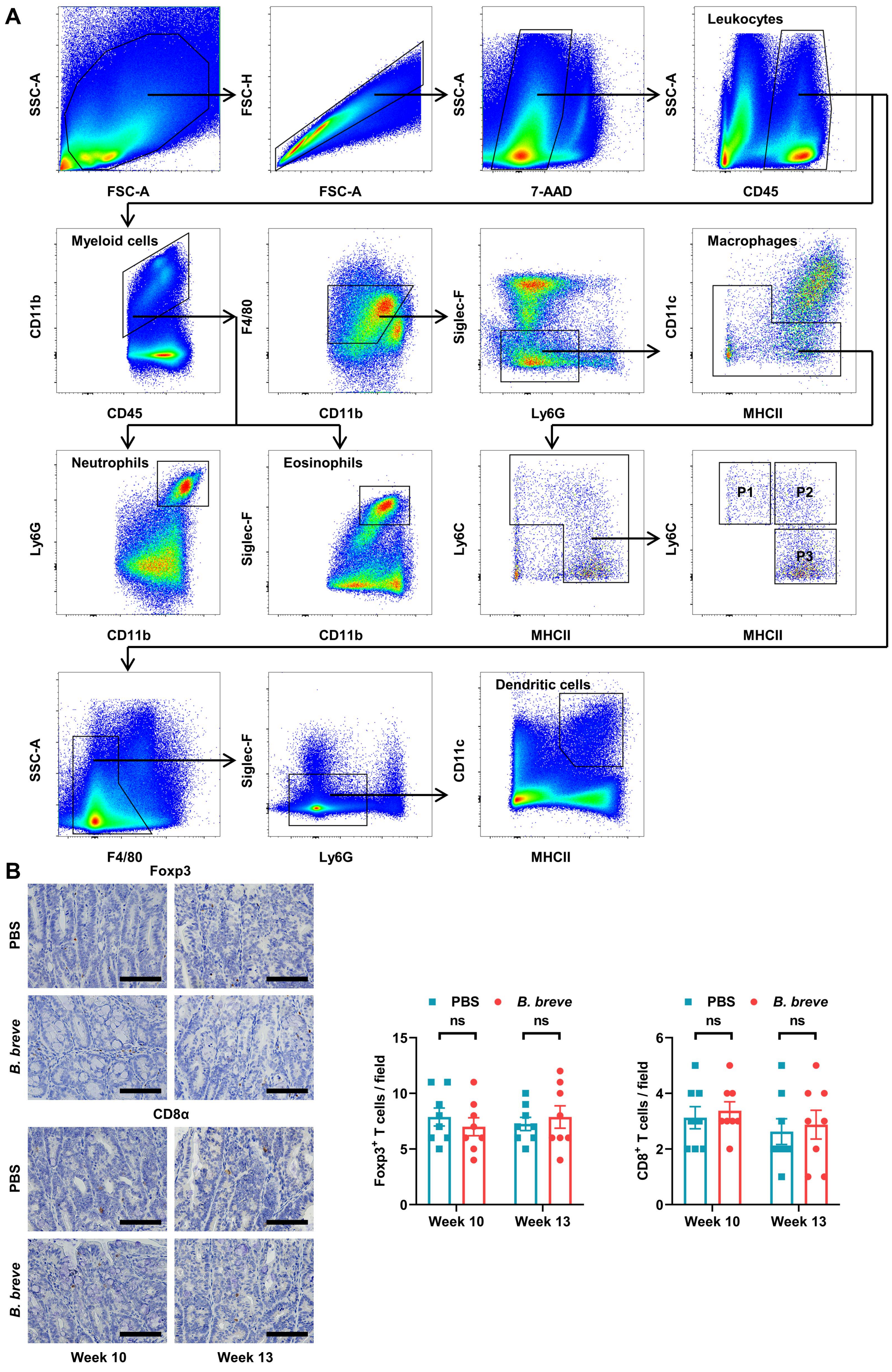

A
SSC-A
FSC-A
FSC-H
7-AAD
Leukocytes
CD45
Myeloid cells
CD11b
F4/80
Siglec-F
Ly6G
Macrophages
CD11c
MHCII
Neutrophils
Eosinophils
Ly6C
P1
P2
P3
Dendritic cells
B
Foxp3
PBS
B. breve
CD8α
Week 10
Week 13
ns
Foxp3+ T cells / field
CD8+ T cells / field
0
5
10
15
2
4
6

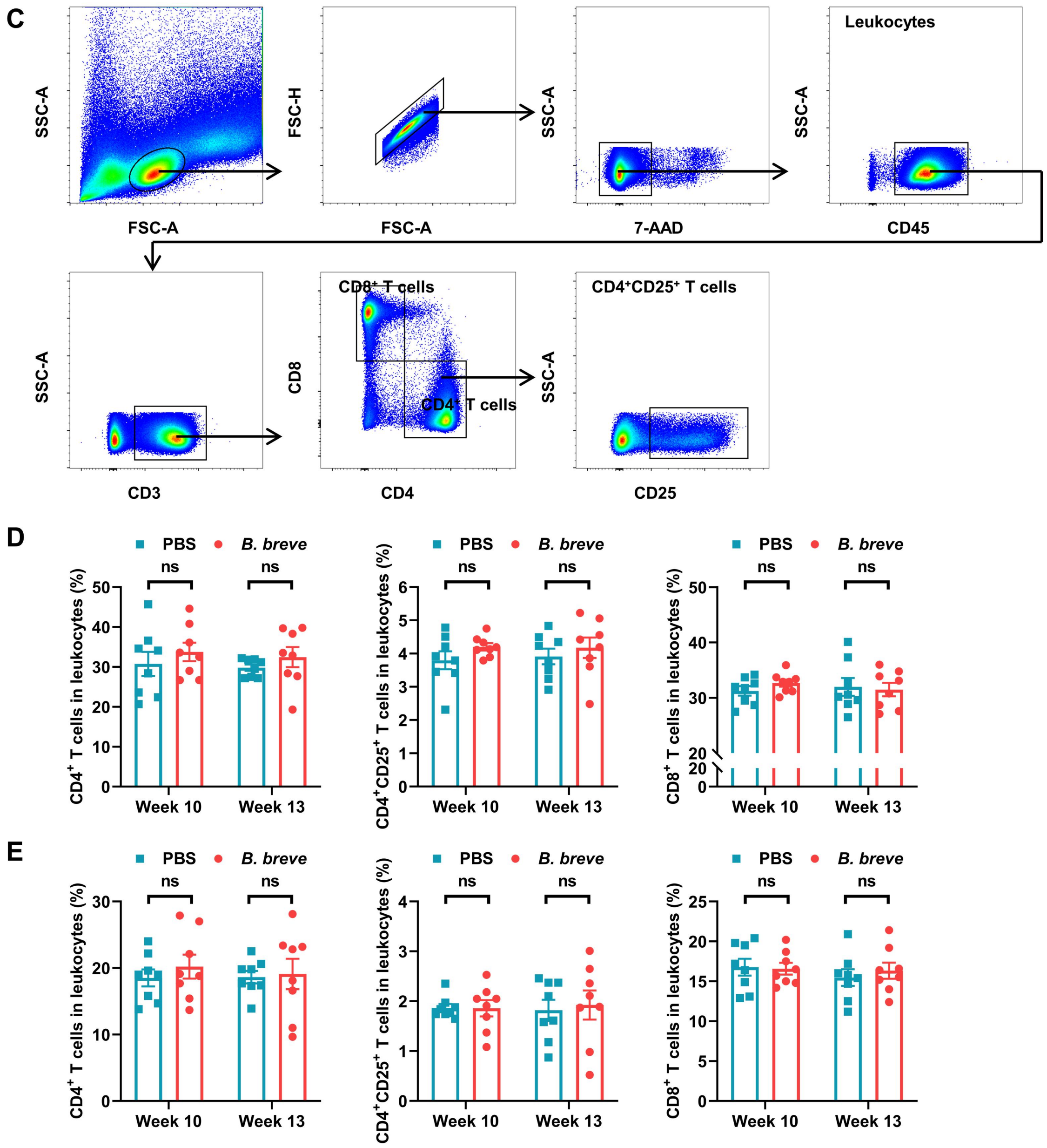

Figure S3: Supplementation with *B. breve lw01* does not affect T cells in CAC mice. (A) Gating patterns used to identify different myeloid cell subsets in the colonic LP. (B) Representative IHC staining and quantitation of Foxp3 and CD8α in the distal colon. Scale bars, 100 µm. (C) Gating patterns used to identify different T cell subsets in the MLN and spleen. (D, E) Proportion of $CD4^+$ T cells, $CD4^+CD25^+$ T cells, and $CD8^+$ T cells in the (D) MLN and (E) spleen assessed by flow cytometry in CAC mice. Data are represented as mean ± SEM. *$P < 0.05$, **$P < 0.01$, ***$P < 0.001$, ns: not significant. *B. breve*: *Bifidobacterium breve*; CAC: colitis-associated colorectal cancer; LP: lamina propria; MLN: mesenteric lymph node.

Figure S4

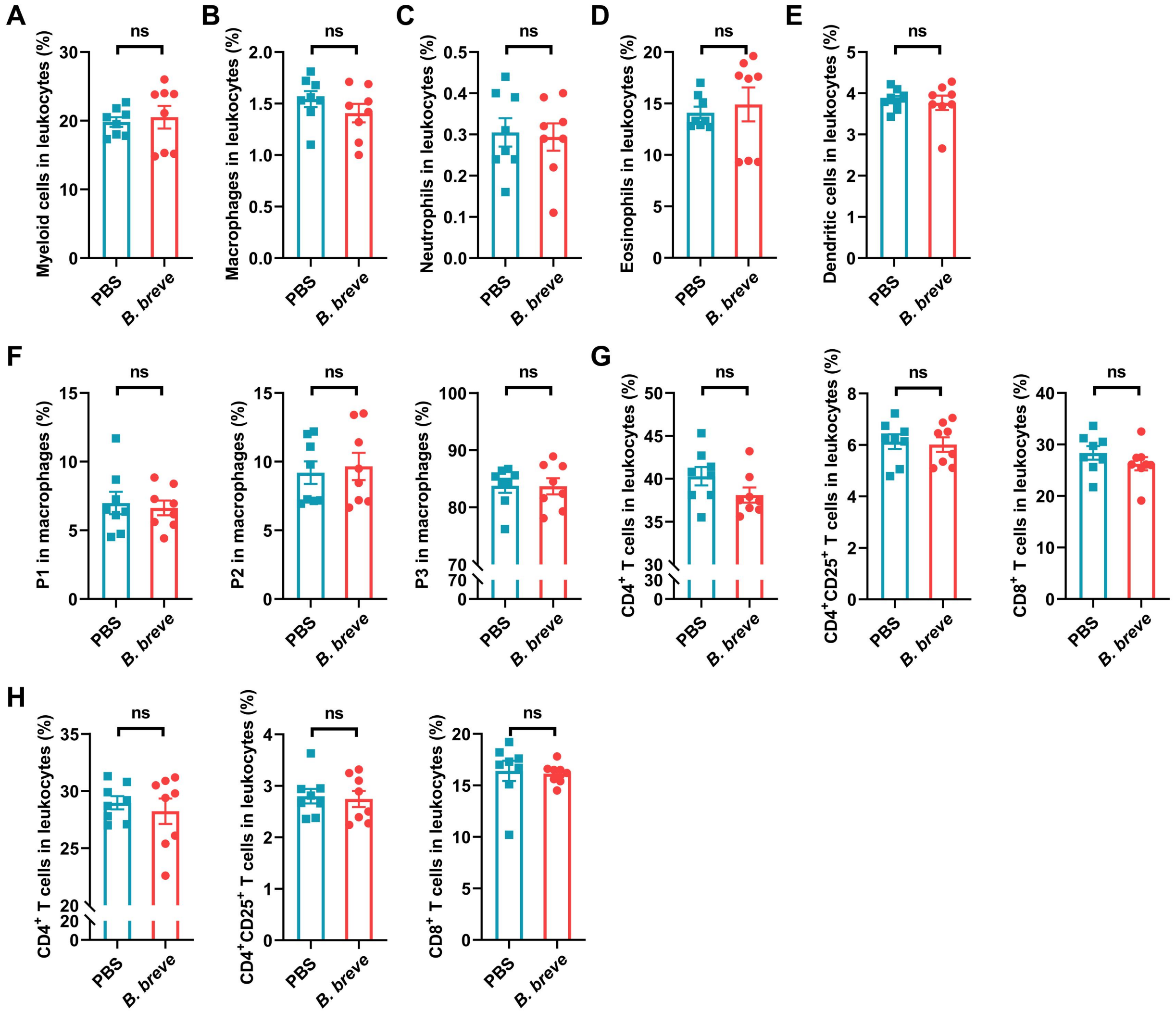

Figure S4: Immune cell subsets are not changed in normal mice. (A-F) Proportion of (A) myeloid cells, (B) macrophages, (C) neutrophils, (D) eosinophils, (E) dendritic cells, and (F) macrophage subsets in the colonic LP assessed by flow cytometry in normal mice. (G, H) Proportion of $CD4^+$ T cells, $CD4^+CD25^+$ T cells, and $CD8^+$ T cells in the (G) MLN and (H) spleen assessed by flow cytometry in normal mice. Data are represented as mean ± SEM. *$P < 0.05$, **$P < 0.01$, ***$P < 0.001$, ns: not significant. *B. breve*: *Bifidobacterium breve*; LP: lamina propria; MLN: mesenteric lymph node; P1: immature colonic macrophage; P3: mature colonic macrophage.

Figure S5

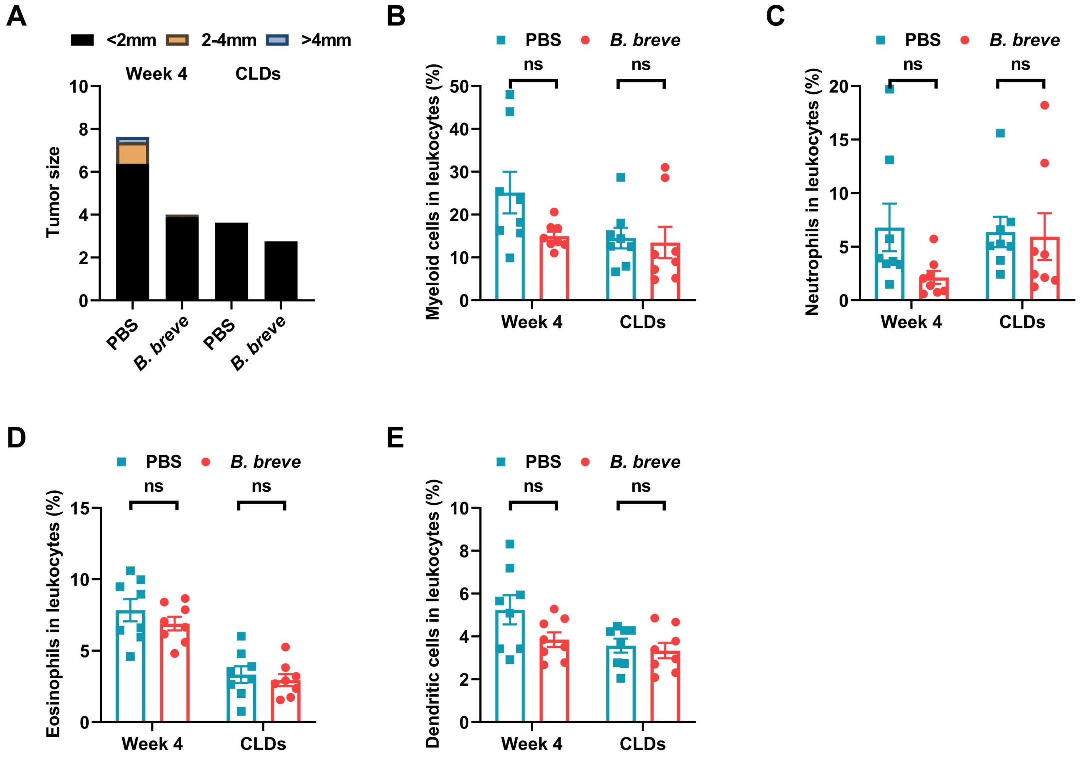

Figure S5: Other myeloid cell subsets in the colonic LP are invariant in macrophage-depleted mice. (A) Statistical histogram of tumor size in CAC mice. (B-E) Proportion of (B) myeloid cells, (C) neutrophils, (D) eosinophils, and (E) dendritic cells in the colonic LP assessed by flow cytometry in CAC mice. Data are represented as mean ± SEM. **P* < 0.05, ***P* < 0.01, ****P* < 0.001, ns: not significant. *B. breve*: *Bifidobacterium breve*; CAC: colitis-associated colorectal cancer; CLD: clodronate liposome; LP: lamina propria.

Figure S6

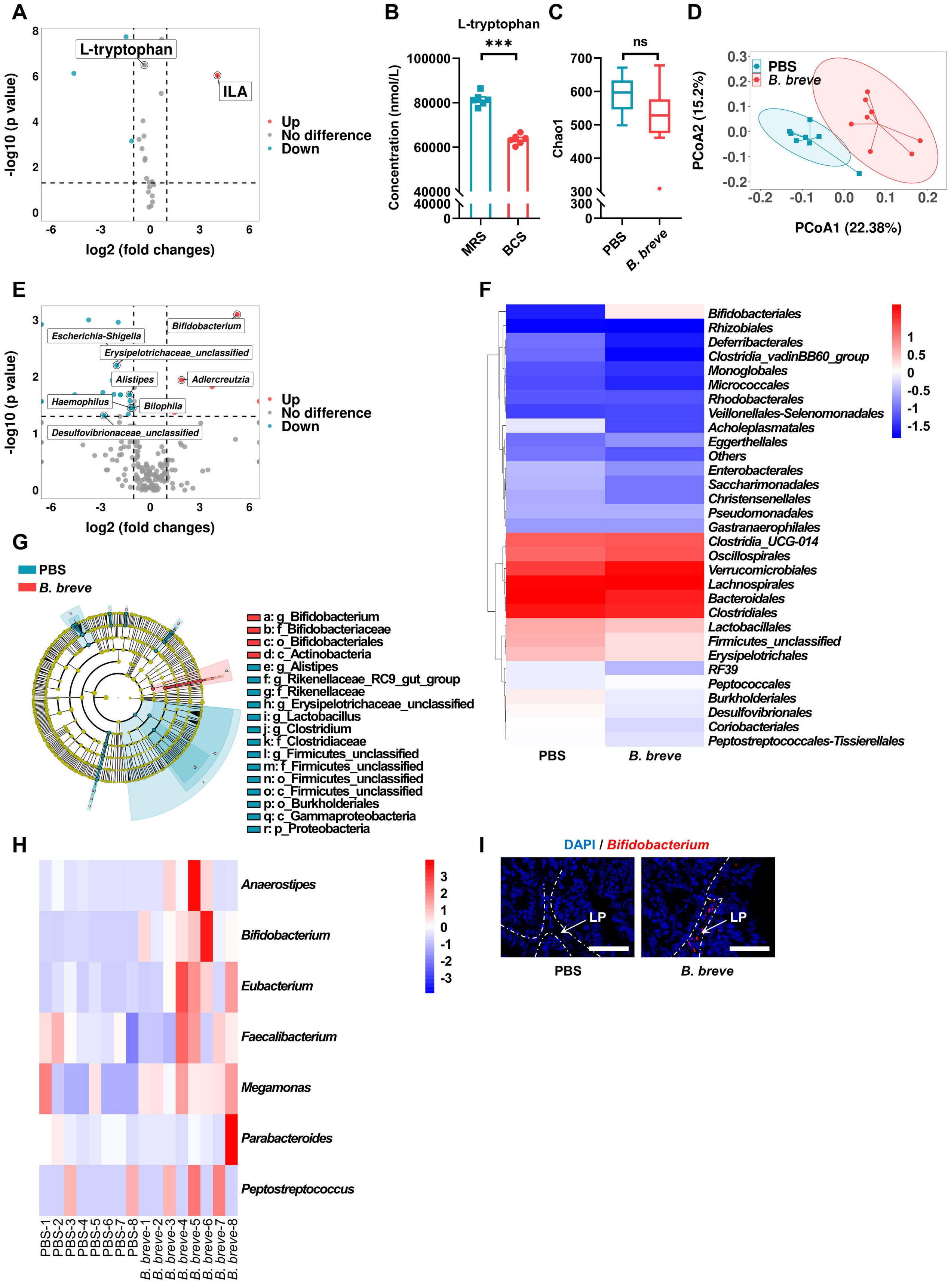

A
L-tryptophan
ILA
-log10 (p value)
log2 (fold changes)
Up
No difference
Down
B
L-tryptophan
***
Concentration (nmol/L)
MRS
BCS
C
ns
Chao1
PBS
B. breve
D
PBS
B. breve
PCoA1 (22.38%)
PCoA2 (15.2%)
E
Bifidobacterium
Escherichia-Shigella
Erysipelotrichaceae_unclassified
Alistipes
Adlercreutzia
Haemophilus
Bilophila
Desulfovibrionaceae_unclassified
-log10 (p value)
log2 (fold changes)
Up
No difference
Down
F
Bifidobacteriales
Rhizobiales
Deferribacterales
Clostridia_vadinBB60_group
Monoglobales
Micrococcales
Rhodobacterales
Veillonellales-Selenomonadales
Acholeplasmatales
Eggerthellales
Others
Enterobacterales
Saccharimonadales
Christensenellales
Pseudomonadales
Gastranaerophilales
Clostridia_UCG-014
Oscillospirales
Verrucomicrobiales
Lachnospirales
Bacteroidales
Clostridiales
Lactobacillales
Firmicutes_unclassified
Erysipelotrichales
RF39
Peptococcales
Burkholderiales
Desulfovibrionales
Coriobacteriales
Peptostreptococcales-Tissierellales
PBS
B. breve
G
PBS
B. breve
a: g_Bifidobacterium
b: f_Bifidobacteriaceae
c: o_Bifidobacteriales
d: c_Actinobacteria
e: g_Alistipes
f: g_Rikenellaceae_RC9_gut_group
g: f_Rikenellaceae
h: g_Erysipelotrichaceae_unclassified
i: g_Lactobacillus
j: g_Clostridium
k: f_Clostridiaceae
l: g_Firmicutes_unclassified
m: f_Firmicutes_unclassified
n: o_Firmicutes_unclassified
o: c_Firmicutes_unclassified
p: o_Burkholderiales
q: c_Gammaproteobacteria
r: p_Proteobacteria
H
Anaerostipes
Bifidobacterium
Eubacterium
Faecalibacterium
Megamonas
Parabacteroides
Peptostreptococcus
PBS-1
PBS-2
PBS-3
PBS-4
PBS-5
PBS-6
PBS-7
PBS-8
B. breve-1
B. breve-2
B. breve-3
B. breve-4
B. breve-5
B. breve-6
B. breve-7
B. breve-8
I
DAPI / Bifidobacterium
LP
PBS
B. breve

Figure S6: *B. breve lw01* modulates gut microbiota of CAC mice and produces ILA. (A) Scatter plot of differential abundant metabolites between *B. breve lw01* and MRS supernatant (Red dots represent upregulated metabolites, and cyan dots represent downregulated metabolites). (B) Concentration of *B. breve lw01*-metabolized L-tryptophan *in vitro*. (C) Microbial richness indices analysis of intestinal microbiota OTUs in the *B. breve* group vs PBS group in CAC mice. (D) Principal coordinate analysis (PCoA) plot of β-diversity to measure the colonic microbial composition in the *B. breve* group vs PBS group in CAC mice. (E) Scatter plot of differential colonic bacteria between the two groups at the genus level (Red dots represent upregulated genus, and cyan dots represent downregulated genus). (F) Heatmap of differential colonic bacteria between the two groups at the order level. (G) Classification tree of different species between the two groups based on LEfSe. (H) Heatmap of bacteria that might produce ILA between the two groups at the genus level. (I) Representative FISH staining with DAPI (blue) and *Bifidobacterium* probe (red) in the distal colon of CAC mice (The arrow indicates the colonic LP). Scale bars, 50 µm. Data are represented as mean ± SEM. **P* < 0.05, ***P* < 0.01, ****P* < 0.001, ns: not significant. *B. breve*: *Bifidobacterium breve*; BCS: the culture supernatant of *B. breve*; CAC: colitis-associated colorectal cancer; FISH: fluorescence *in situ* hybridization; ILA: indole-3-lactic acid; LP: lamina propria; MRS: de Man, Rogosa and Sharpe medium; OTU: operational taxonomic unit; PCoA: principal coordinate analysis.

Figure S7

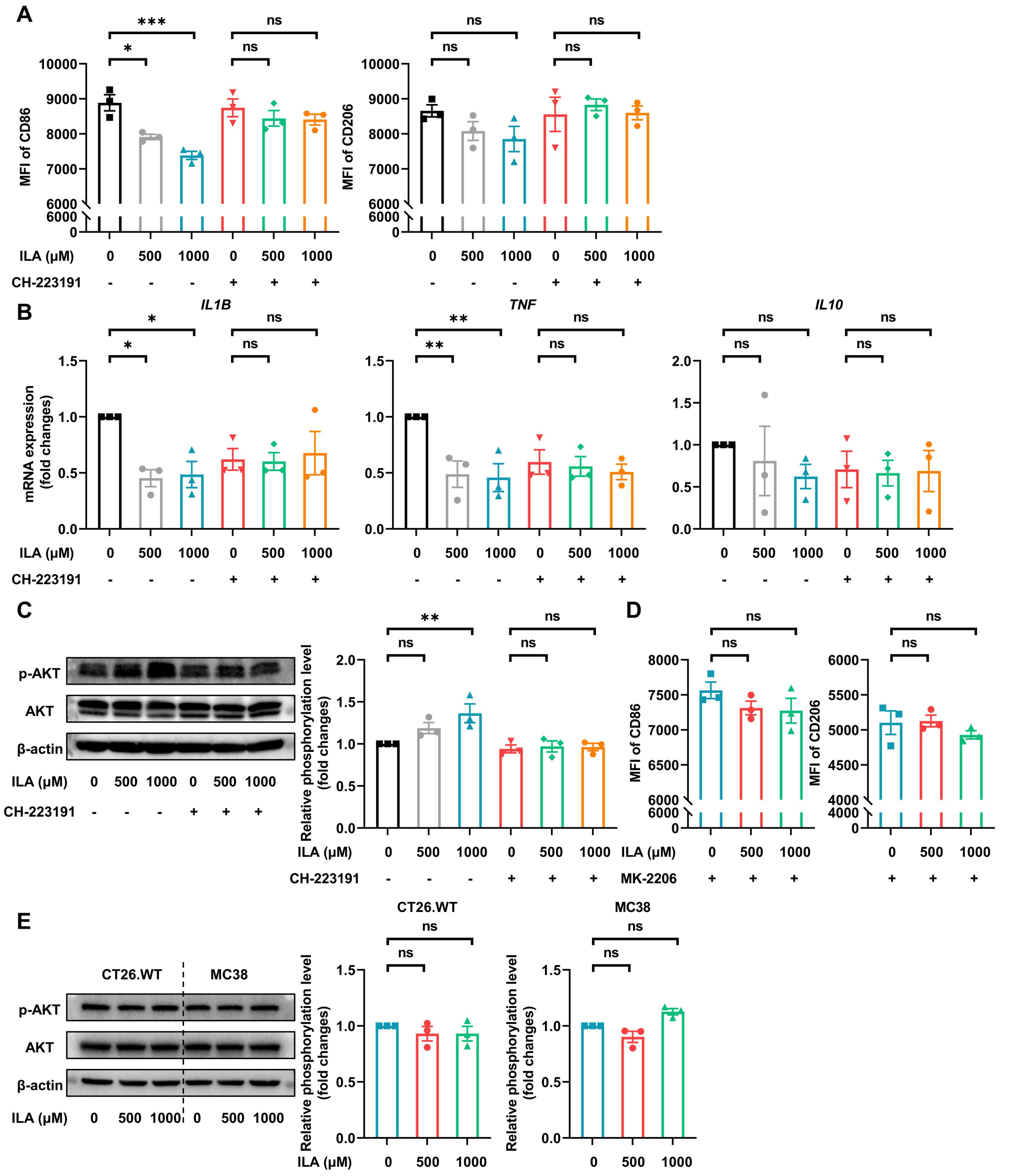

Figure S7: ILA alleviates LPS-induced pro-inflammatory response of macrophages derived from hPBMCs via the PI3K/AKT signaling pathway. (A) Effect of ILA with or without CH-223191 on the proportion of CD86 or CD206 in macrophages derived from hPBMCs, as tested by flow cytometry. (B) Gene expression of *IL1B*, *TNF* and *IL10* in macrophages derived from hPBMCs. (C) Representative Western blot images and statistical histogram of AKT phosphorylation levels in macrophages derived from hPBMCs. (D) Effect of ILA with or

without MK-2206 on the proportion of CD86 or CD206 in macrophages derived from hPBMCs, as tested by flow cytometry. (E) Representative Western blot images and statistical histogram of AKT phosphorylation levels in CT26.WT and MC38 cell lines. Data are represented as mean ± SEM. *P < 0.05, **P < 0.01, ***P < 0.001, ns: not significant. hPBMC: human peripheral blood mononuclear cell; ILA: indole-3-lactic acid; LPS: lipopolysaccharide; MFI: mean fluorescence intensity.

Figure S8

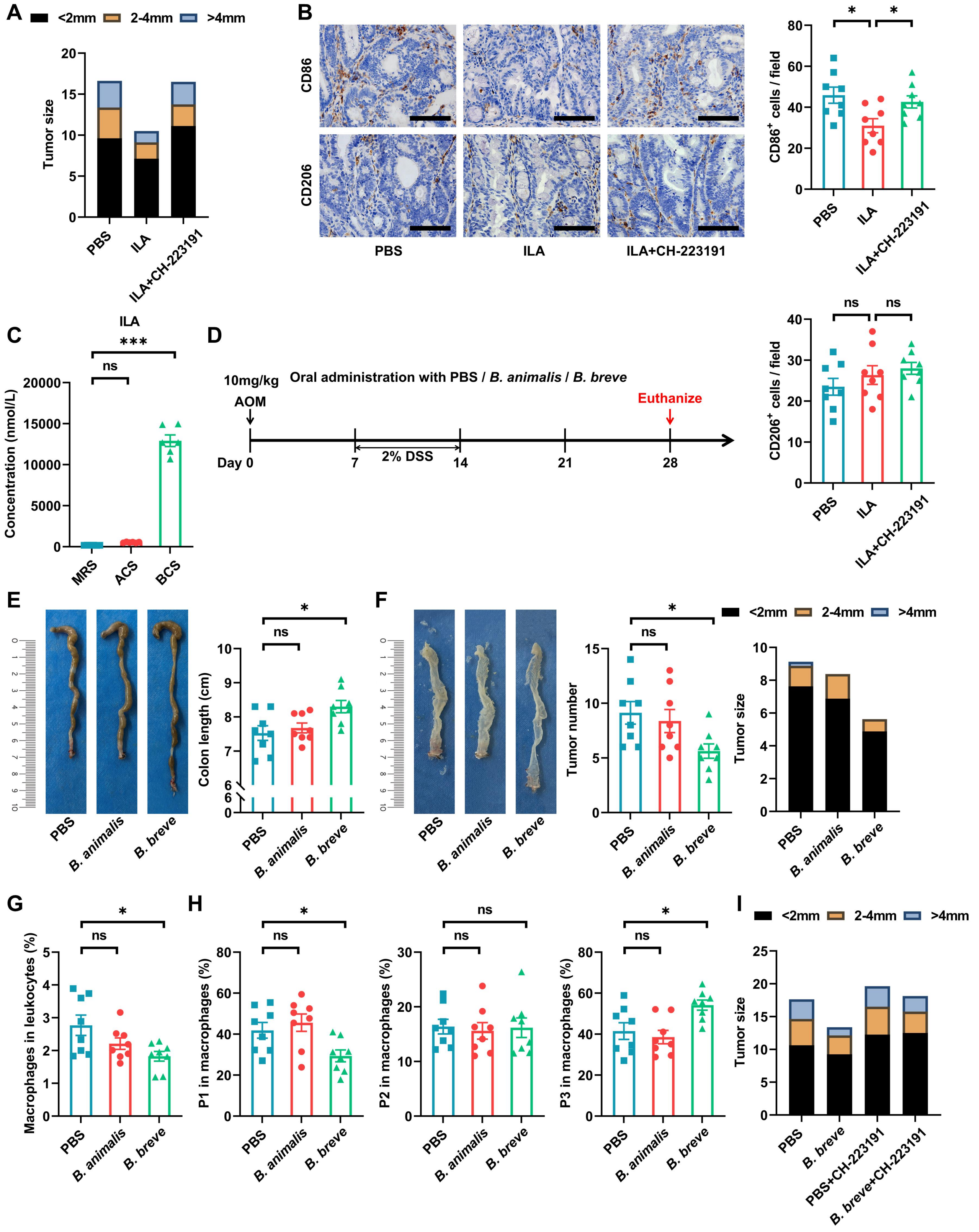

Figure S8: *B. animalis CICC 24672* could not produce ILA to exert the anti-inflammation and anti-tumor effects in CAC mice. (A) Statistical histogram of tumor size in CAC mice. (B) Representative IHC staining and quantitation of CD86 and CD206 in the distal colon. Scale bars, 100 μm. (C) Concentration for ILA production of

B. animalis CICC 24672 and *B. breve lw01* metabolizing L-tryptophan *in vitro*. (D) Schematic diagram for establishing CAC models induced by AOM/DSS in three groups (PBS, *B. animalis CICC 24672*, or *B. breve lw01* was administered daily during CAC development). n=8 per group. (E) Representative colonic images and statistical histogram of colon length. (F) Representative colonic images and statistical histogram of tumor number and size. (G, H) Proportion of (G) macrophages and (H) macrophage subsets in the colonic LP assessed by flow cytometry in CAC mice. (I) Statistical histogram of tumor size in CAC mice. Data are represented as mean ± SEM. *$P < 0.05$, **$P < 0.01$, ***$P < 0.001$, ns: not significant. ACS: the culture supernatant of *B. animalis*; AOM: azoxymethane; *B. animalis*: *Bifidobacterium animalis*; *B. breve*: *Bifidobacterium breve*; BCS: the culture supernatant of *B. breve*; CAC: colitis-associated colorectal cancer; DSS: dextran sodium sulfate; ILA: indole-3-lactic acid; LP: lamina propria; MRS: de Man, Rogosa and Sharpe medium; P1: immature colonic macrophage; P3: mature colonic macrophage.

Figure S9

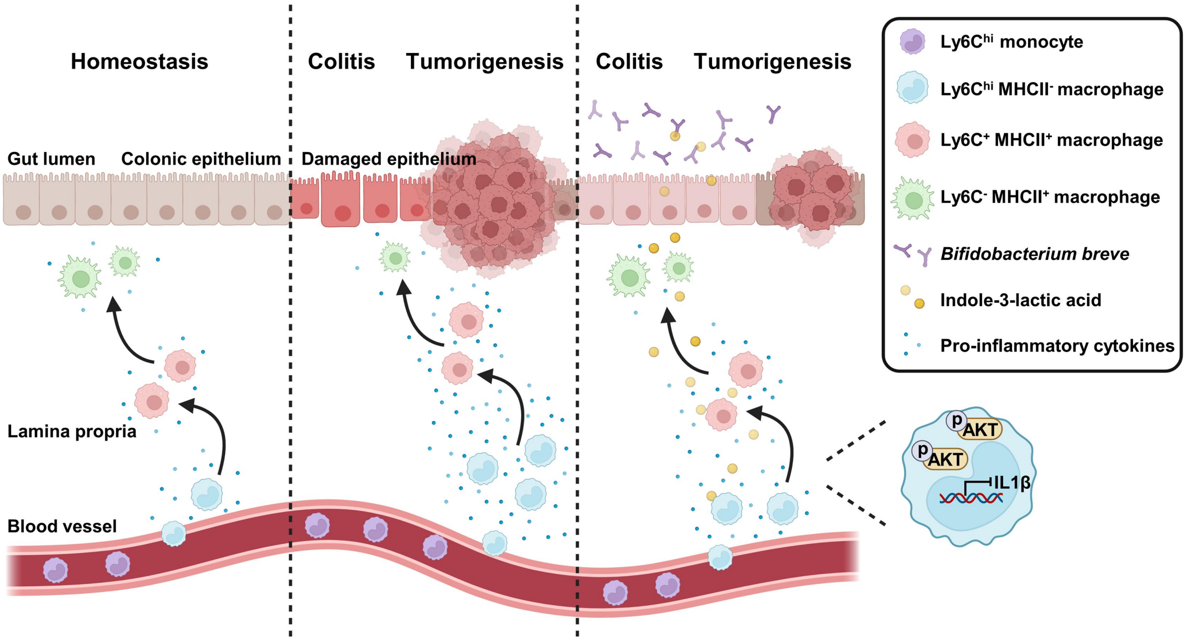

Figure S9: Schematic overview of the *B. breve lw01*-macrophage crosstalk during CAC tumorigenesis. Left: Most colonic macrophages are replenished by Ly6C^{hi} circulating monocytes. As these Ly6C^{hi} monocytes enter the lamina propria, they gradually differentiate into mature colonic macrophages (Ly6C^{-}MHCII^{+}) to maintain intestinal homeostasis. Middle: The conventional process is disrupted by the inflammatory intestinal microenvironment, resulting in a massive accumulation of immature colonic macrophages (Ly6C^{hi}MHCII^{-}). Conditional pathogenic bacteria with their metabolites continuously activate immature colonic macrophages, further aggravating chronic inflammation and leading to tumorigenesis. Right: *Bifidobacterium breve* ameliorates the precancerous inflammatory intestinal milieu to inhibit tumorigenesis by directing the differentiation of immature colonic macrophages, which is attributed to indole-3-lactic acid, alleviating the pro-inflammatory response of macrophages via the PI3K/AKT signaling pathway.
